# Uncertainties about the benefit-risk balance of oncology medicines assessed by the European Medicines Agency

**DOI:** 10.1016/j.esmoop.2024.103991

**Published:** 2024-12-09

**Authors:** A.C. Taams, C.A. Herberts, A.C.G. Egberts, N. Zafiropoulos, F. Pignatti, L.T. Bloem

**Affiliations:** 1 Division of Pharmacoepidemiology and Clinical Pharmacology, Utrecht Institute for Pharmaceutical Sciences, Utrecht University, Utrecht, The Netherlands; 2 Dutch Medicines Evaluation Board, Utrecht, The Netherlands; 3 Department of Clinical Pharmacy, University Medical Centre Utrecht, Utrecht, The Netherlands; 4 European Medicines Agency, Amsterdam, The Netherlands

**Keywords:** oncology medicines, uncertainties, classification, regulatory decision making, European Medicines Agency

## Abstract

**Background:**

Drug regulators assess and describe uncertainties regarding treatment outcomes and the benefit-risk balance of newly authorised medicines. We aimed to evaluate the type and number of uncertainties described in the benefit-risk assessment for initial marketing authorisations of oncology medicines assessed by the European Medicines Agency (EMA). We also aimed to develop a systematic classification of uncertainties to contribute to improved communication about uncertainties.

**Materials and methods:**

We included all medicines containing a new active substance assessed by the EMA and granted an initial marketing authorisation by the European Commission in 2011-2022 for an oncology indication. We extracted characteristics of these oncology medicines and uncertainties described under the benefit-risk balance section of European public assessment reports. Uncertainties were categorised and their frequencies stratified according to time of marketing authorisation, and medicine and regulatory characteristics.

**Results:**

In total, 121 oncology medicines were included for which 800 (median 6, range 0-23) uncertainties were identified. Uncertainties were classified into five categories: safety (*n* = 404, 51%), efficacy (*n* = 322, 40%), pharmacology (*n* = 58, 7%), use in clinical practice (*n* = 10, 1%), and quality (*n* = 6, 1%). Among 27 subcategories, most uncertainties were related to specific adverse events (*n* = 156, 20%), effect size (*n* = 155, 20%), safety in subpopulations (*n* = 124, 16%), or efficacy in subpopulations (*n* = 88, 11%). The type of medicine (*P* = 0.012), type of marketing authorisation (*P* = 0.001), and year of marketing authorisation (*P* = 0.007) were associated with the number of uncertainties per medicine, with the highest number observed for cell and gene therapies [8 (3-23)], medicines granted conditional marketing authorisation [7 (3-23)], and medicines authorised in 2019-2022 [7 (2-23)].

**Conclusion:**

At the time of initial marketing authorisation of oncology medicines, uncertainties about their benefit-risk balance most often concerned safety aspects, followed by efficacy. The number of uncertainties was highest for cell and gene therapies, conditionally authorised medicines, and medicines authorised in recent years.

## Highlights

• For 121 novel oncology medicines, a median of 6 (range 0-23) uncertainties were identified.
• These mainly related to safety (51%) and efficacy (40%), and only a few to quality and use in clinical practice.
• Subcategories mainly concerned specific adverse events, effect size, and safety or efficacy in subpopulations.
• More uncertainties were observed for cell and gene therapies, and more recently and conditionally authorised medicines.

## Introduction

The European Medicines Agency (EMA) is the medicine regulatory authority of the European Union. The agency is responsible for evaluation, supervision, and monitoring of a medicine’s benefit-risk balance throughout its lifecycle, for which assessments are carried out by experts of national authorities and the EMA’s committee members.^1^ The regulatory review process starts at the application for marketing authorisation and continues post-authorisation, with regulators assessing all data related to the medicine’s quality, safety, and efficacy. However, regulatory decision making on medicines can be challenging due to incompleteness of knowledge and evidence, causing uncertainty.^2^ Unmet medical needs have also fuelled alternative types of marketing authorisation, such as conditional marketing authorisation and marketing authorisation under exceptional circumstances, which allow less comprehensive data at initial authorisation and, consequently, a higher level of uncertainty.^3^^,^^4^

While uncertainties can emerge at different stages of a medicine’s lifecycle, regulatory authorities specifically identify and reflect upon them during the initial marketing authorisation process. The uncertainties are explicitly described in the European public assessment report (EPAR). Assessors are guided by a template that discusses the need to describe which key findings and uncertainties should be part of the medicine’s benefit-risk assessment in the EPAR.^5^ However, the current guidance does not provide a consistent method for describing certain uncertainties among different medicines. Verweij et al.^6^ noted inconsistencies in wording, length, and detail of how uncertainties are described.

It is important for clinicians to understand the medicine regulatory processes, the data supporting the marketing authorisation, and the uncertainties about the treatment outcomes. However, there seems to be a gap between what clinicians want to know and what is communicated to them.^7^, ^8^, ^9^, ^10^ Davis et al. revealed that regulatory sources of information, such as patient information leaflets, often lack information on uncertainties and available evidence.^11^ To support optimal clinical decision making, it is essential to improve understanding of uncertainties at the time of marketing authorisation and throughout a medicine’s lifecycle, as uncertainties may persist and evolve.

The need to understand uncertainties identified by regulators may be even more important for oncology medicines, since a substantial part of medicines under development as well as those recently authorised are intended for oncology indications. Oncology is also the domain with relatively many new concepts such as novel mechanisms of action (e.g. CAR-T-cell therapies), non-randomised study designs (e.g. single-arm trials, external control designs), and patient selection (e.g. tumour agnostic), which can complicate the assessment of the benefit-risk balance by regulators and may result in remaining uncertainties.^12^, ^13^, ^14^ However, little is known about the uncertainties about the benefit-risk balance of medicines at the time of marketing authorisation, both for oncology and non-oncology medicines. Therefore, we aimed to evaluate the type and number of uncertainties about the benefit-risk balance of oncology medicines as assessed by the EMA at the time of initial marketing authorisation in the European Union. In doing so, we also aimed to develop a systematic classification of these uncertainties to contribute to improved communication about uncertainties among stakeholders involved in drug development and evaluation, including drug regulatory authorities, pharmaceutical companies, health technology assessment bodies, clinicians, and patients.

## Materials and methods

### Study design, cohort selection, and data source

We carried out a retrospective cohort study that included all initial marketing authorisation applications of medicines (i) with a new active substance, defined as not already authorised or used in another medicine, (ii) with an oncology indication, and without indications for benign tumours, precancerous conditions, diagnostics or therapeutic radiopharmaceuticals, and that were (iii) authorised by the European Commission (EC) (iv) between 01 January 2011 and 31 December 2022 (iv) based on a complete dossier [i.e. legal basis referring to Article 8(3) of Directive 2001/83/EC^15^]. Medicines based on different dossiers such as generic or hybrid applications were excluded. The medicines were identified via the EC’s Union Register of medicines for human use.^16^ The data sources used for this study were the EPARs for each medicine, through which the scientific assessments of the Committee for Medicinal Products for Human Use is made publicly available by the EMA.^17^

### Uncertainties: identification and classification

For each included oncology medicine, remaining uncertainties at the time of initial marketing authorisation are described in the specific uncertainty sections of the EPARs (i.e. on the favourable and unfavourable effects). These sections have been included in the EPAR template since mid-2010.^6^ Given the absence of a clear definition, we defined an uncertainty as a text fragment within the EPAR’s uncertainty sections that describes a specific concern related to the effects of the medicine within its authorised indication. Therefore, uncertainties related only to specific subpopulations that were excluded by restricting the indication were disregarded. One sentence could contain multiple uncertainties. All uncertainties were extracted by ACT. After identification of uncertainties, inductive reasoning was used to create mutually exclusive uncertainty categories for development of our classification system. When a text fragment was considered to be related to multiple categories of uncertainties, one category was considered most important and used for classification, following a predefined hierarchy (see Box A1, Rules for the classification of uncertainties). Initially, ACT and LTB discussed all uncertainties that were difficult to classify, until consensus was reached. If no consensus could be reached, uncertainties were also discussed with CAH and ACG until consensus was reached. In addition, we carried out blinded validation rounds between ACT, LTB, ACG, and CAH, after which uncertainties were recategorised when considered necessary.

### Medicine characteristics

We categorised oncology medicines as small molecules, biologicals, or cell and gene therapies (CGTs) based on the classification by drugbank.com. Although CGTs are by definition also biologicals, this study reflected these as a separate category due to their distinct nature, which is also supported by the separate legislation for the medicine type.^18^ By evaluation of ATC codes, we categorised medicines also in therapeutic classes (signal transduction inhibitors, CGTs, combination therapies, or other oncology medicines).

To assess whether medicines were first-in-class, we identified their primary target based on pharmacological subgroups of the ATC code (i.e. the first five characters). If a distinct pharmacological subgroup was not available (e.g. L01EX ‘Other protein kinase inhibitors’ and L01XL ‘Antineoplastic cell and gene therapy’), we consulted EPARs to identify the primary target. Medicines with closely related targets, such as programmed cell death protein 1 and programmed death-ligand 1 inhibitors, vascular endothelial growth factor (VEGF) and VEGF receptor inhibitors, and microtubule destabilisers with different binding sites, were considered to have the same target. For each target, the first medicine authorised in the EU was considered first-in-class, as well as any medicines authorised within 3 months after authorisation of the first medicine, thereby acknowledging that these medicines had largely undergone regulatory assessment in parallel. In case a medicine contained more than one active substance, we considered it first-in-class if at least one active substance was aimed at a new target. We disregarded active substances that did not contribute to the primary therapeutic action of a medicine, such as pharmacokinetic enhancers for pyrimidine analogues.

### Regulatory characteristics

Regulatory characteristics concern the type of marketing authorisation (standard, conditional, or exceptional), the use of a regulatory support or expedited programme, and orphan designation at the time of initial marketing authorisation. A conditional marketing authorisation can be granted when clinical data are not yet ‘comprehensive’ but indicate that the benefit-risk balance is positive, the medicine is expected to fulfil an unmet medical need, comprehensive clinical data are expected to be submitted post-authorisation, and the benefits of availability outweigh the risks of lacking comprehensive data.^4^^,^^19^ A marketing authorisation under exceptional circumstances can be granted if clinical data are non-comprehensive and it is not possible to collect comprehensive efficacy and safety data under normal conditions of use, for example, if the disease is rare or because collection of these data is unethical, or because scientific knowledge is incomplete.^20^ In addition, we identified whether medicines underwent expedited and regulatory support programmes, comprising accelerated regulatory assessment (150 days instead of 210 days of assessment time by the EMA) and PRIority MEdicines (PRIME) support, which is facilitated by the EMA to enhance drug developmental and regulatory support for medicines that target unmet medical needs. Finally, we considered the designation of orphan status at the time of initial authorisation.

### Data management, analysis, and statistics

We assessed whether there were differences in the number of uncertainties between categories of (i) time, (ii) medicine, and (iii) regulatory characteristics. We first assessed if the data were normally distributed by carrying out the Kolmogorov–Smirnov test. Since our data were not normally distributed, we carried out two non-parametric statistical tests: (i) The Mann–Whitney *U* (MWU) test for characteristics comprising two categories and (ii) the Kruskal–Wallis (KW) test for characteristics comprising more categories. Results were reflected as the median (range) number of uncertainties and the relevant *P* value per characteristic. We created a model with all potential predictors and then removed variables from the model one by one until we found the most parsimonious model, namely the one with the aforementioned three variables. A multiple linear regression analysis was carried out to examine the independent association between the potential predictors, i.e. time of authorisation, medicine and regulatory characteristics, and the number of uncertainties per medicine. Based on the results of the regression analysis, a Venn diagram was made to visualise how the characteristics in our dataset contributed to the number of uncertainties per medicine. For data extraction and management, Microsoft Excel was used. Descriptive and statistical analyses were carried out in SPSS. R studio and Adobe Illustrator were used to create figures.

## Results

### Study cohort characteristics

In the 12-year study period from 2011 to 2022, the EC authorised 1001 medicines following positive assessment by the EMA, of which 400 (40%) contained a new active substance. Among these, 121 (30%) were indicated for an oncology indication and authorised based on a complete dossier (Supplementary Figure S1 and Table S1, available at https://doi.org/10.1016/j.esmoop.2024.103991), and thus included in our study. Of these 121 medicines, the majority comprised small molecules (*n* = 73, 60%), followed by biologicals (*n* = 39, 32%) and CGTs (*n* = 9, 7%) (Table 1). Concerning therapeutic classes, signal transduction inhibitors were predominant (*n* = 82, 68%). Forty-four (*n* = 44, 36%) oncology medicines were considered first-in-class.

**Table 1:** Characteristics of the study cohort

	Medicines cohort, *n* (%)
**Total cohort**	121 (100)
**Median**	—
**Year of marketing authorisation**	
2011-2014	33 (27)
2015-2018	42 (35)
2019-2022	46 (38)
**Medicine characteristics**	
Medicine type	
Small molecules	73 (60)
Biologicals	39 (32)
Cell and gene therapies	9 (7)
Therapeutic class (by ATC code)	
Signal transduction inhibitors	82 (68)
Tyrosine kinase inhibitors	40 (33)
Monoclonal antibodies	35 (29)
Other Signal transduction inhibitors	7 (6)
Cell and gene therapies	9 (7)
Combination therapies	3 (2)
Other oncology medicines	27 (22)
First-in-class	
Yes	44 (36)
No	77 (64)
**Regulatory characteristics**	
EMA type of marketing authorisation	
Standard MA	78 (64)
Conditional MA	41 (34)
Exceptional MA	2 (2)
Expedited and support programmes	
Yes	20 (17)
Accelerated assessment	13 (11)
PRIME support	9 (7)
No	101 (83)
Orphan medicine	
Yes	46 (38)
No	75 (62)

Therapeutic classes were defined by ATC code. As such, ‘other oncology medicines’ included antimetabolites (L01BC), anti-hormones (L02BX, L02BB), antimitotic agents (L01CD), PARP inhibitors (L01XK), CDK-46 inhibitors (L01EF), antitumour antibiotics (L01DB), and other oncolytics (L01XX, L04AX, L01XH, L01XD, L01XJ).

ATC, anatomical therapeutic chemical; CDK, cyclin-dependent kinases; EMA, European Medicines Agency; MA, marketing authorisation; PARP, poly-ADP ribose polymerase; PRIME, priority medicines.

Regarding type of marketing authorisations, a standard marketing authorisation was most common (*n* = 78, 65%), followed by conditional marketing authorisation (*n* = 41, 34%) and marketing authorisation under exceptional circumstances (*n* = 2, 2%). With respect to expedited and regulatory support programmes, 13 medicines underwent accelerated assessment (11%). Also, between 2018 and 2022, nine medicines benefited from PRIME support (*n* = 9, 7%). Forty-six medicines received an orphan designation (*n* = 46, 38%).

### Classification of the uncertainties

**Figure 1:**
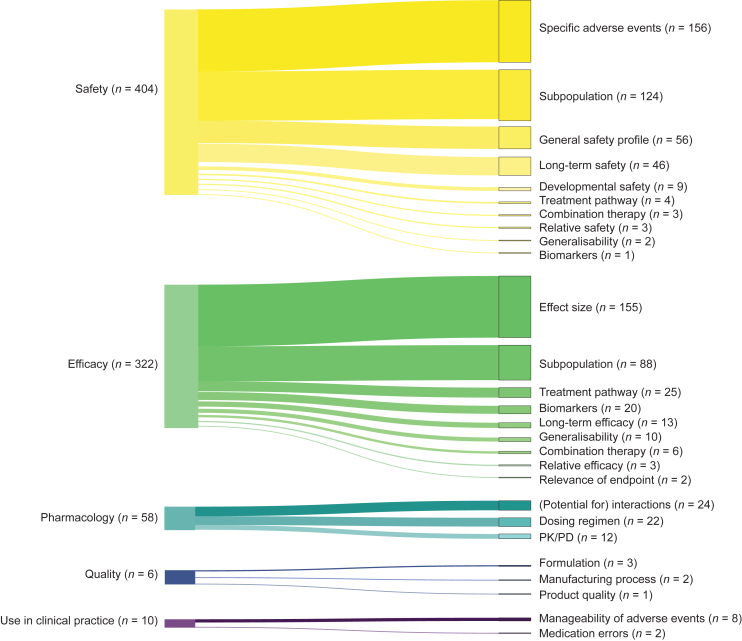
**Frequencies of uncertainties per main category and related subcategories.** PK/PD, pharmacokinetics/pharmacodynamics.

In total, we identified 808 uncertainties, of which eight were excluded since these were mitigated at the time of marketing authorisation by restricting the indication of the medicine, leaving 800 uncertainties that we included in our study (median 6, range 0-23 per medicine). An overview of the classification of these uncertainties, including definitions and examples, is shown in Table 2. The 800 uncertainties pertained to five main categories: safety [*n* = 404, 51%; median 3 (range 0-18) per medicine], efficacy [*n* = 322, 40%; 2 (0-14)], pharmacology [*n* = 58, 7%; 0 (0-3)], use in clinical practice [*n* = 10, 1%; 0 (0-4)], and quality [*n* = 6, 1%; 0 (0-1)]. Supplementary Figure S2, available at https://doi.org/10.1016/j.esmoop.2024.103991, shows the distribution of uncertainties in the five main categories for each year between 2011 and 2022. Within the main categories, uncertainties were further categorised into 27 more specific subcategories. The four most common subcategories for safety-related uncertainties were ‘specific adverse events’ [*n* = 156, 20%; 1.0 (0-10)], ‘safety in subpopulations’ [*n* = 124, 16%; 0 (0-12)], ‘general safety profile’ [*n* = 56, 7%; 0 (0-4)], and ‘long-term safety’ [*n* = 46, 6%; 0 (0-2)]. The four most common subcategories for efficacy-related uncertainties were ‘effect size’ [*n* = 155, 20%; 1.0 (0-5)], ‘efficacy in subpopulations’ [*n* = 88, 11%; 1.0 (0-4)], ‘treatment pathway’ [*n* = 25, 3%; 0 (0-1)], and ‘biomarkers’ [*n* = 20, 3%; 0 (0-2)]. The frequencies of uncertainties for each main category and related subcategories are shown in Figure 1. An overview of the uncertainty classifications, definitions and examples is shown in Table 2.

**Table 2:** Classification system for uncertainties

Category of uncertainty	Subcategory of uncertainty	Definition of uncertainty per classification Examples of uncertainties from EPARs as ‘direct quotes’ (active substance, brand name, year of authorisation)
Efficacy	Effect size: including, e.g. magnitude of effect, duration of response, time-to-event, quality of life	Uncertainty concerning the size of the medicine’s efficacy effect estimate 1. “The design of the study with no comparative arm is of concern because the ORR, PFS, and OS data cannot be directly compared to other treatment results in the same population.” (Daratumumab, Darzalex, 2016) 2. “For 1^st^ line treatment, while ORR and DOR appear promising, more mature PFS data are required from Part B in order to better estimate patient benefit. Data from the final analysis are required to confirm the magnitude of the effect of the treatment in terms of PFS, but also in terms of possible patient survival.” (Avelumab, Bavencio, 2017) 3. “A clinically relevant difference could be observed in the duration of response across JCAR017 studies, with subjects in US study 017001 faring better than EU patients in study BCM-001. The reasons behind the lower response rate and shorter response duration observed in phase II study BCM-001 are not completely understood, although limited numbers do not exclude the possibility of random findings.” (Lisocabtagene maraleucel, Breyanzi, 2022)
	Subpopulation:including, e.g. elderly or patients with hepatic or renal impairments	Uncertainty concerning the medicine’s efficacy in a subpopulation 4. “No data are available regarding the efficacy of the drug in races other than whites (blacks, Asian, etc.).” (Trametinib, Mekinist, 2014) 5. “A number of uncertainties were identified during the assessment, including the specific population included in the pivotal trial (co-existing medical conditions and/or renal impairment).” (Obinutuzumab, Gazyvaro, 2014) 6. “Efficacy results appear to be consistent regardless of BRCA positive status; however, due to the small number of patients with BRCA positive status (*n* = 43; 8.1%) no firm conclusions can be drawn from these results. Information on BRCA mutational status was lacking for 35% of study population.” (Sacituzumab govitecan, Trodelvy, 2021)
	Treatment pathway: including, e.g. previous treatment re-treatment	Uncertainty concerning the positioning of the medicine in the treatment pathway with regard to efficacy 7. “There is a general uncertainty on the appropriate positioning of talazoparib in a treatment context where platinum based chemotherapy is an option.” (Talazoparib, Talzenna, 2019) 8. “Even though a trend in favour of the Ipd arm was observed at the interim analysis, the trend towards longer OS should be interpreted in the context of the subsequent therapy. Final OS analysis, including subgroup analyses by best response to previous therapy and by refractoriness to lenalidomide, PI inhibitors or both, from study EFC14335 will be submitted in line with the CHMP recommendation.” (Isatuximab, Sarclisa, 2020) 9. “The B/R of retreatment with ide-cel is uncertain, as the responses reported in the re-treated population were infrequent (5 PR and one VGPR reported in 29 retreated patients in study MM-001), and a limited PFS was observed.” (Idecabtagene vicleucel, Abecma, 2021)
	Biomarkers	Uncertainty concerning associations between potential biomarkers and the medicine’s efficacy outcomes^25^ 10. “The role of the biomarkers PD-L1 or PD-L2 expression as potential predictive or prognostic biomarkers remains undetermined.” (Nivolumab, Opdivo, 2015) 11. “In addition, genomic analysis of baseline samples remaining after centralized BRAF testing would be informative to assess whether there is a relationship between baseline mutations and efficacy outcomes.” (Encorafenib, Braftovi, 2018)
	Relevance of endpoint	Uncertainty concerning the relevance of the endpoints used in clinical trials with the medicine 12. “Despite ORR is a commonly used endpoint in oncology studies, its use is usually limited to exploratory studies since it is not able to reliably estimate the ultimate benefit for patients in terms of life expectancy.” (Osimertinib, Tagrisso, 2016) 13. “Given the limitations associated with the uncontrolled single-arm design of the pivotal study and that the primary endpoint, CRR, is not an established surrogate endpoint in r/r FL, sufficiently mature DOR data are important.” (Mosunetuzumab, Lunsumio, 2022)
	Combination therapy	Uncertainty concerning the medicine’s efficacy in combination with another medicine 14. “In order to further evaluate the efficacy of Tecentriq and provide further confirmation of the efficacy assumptions in 1L UC patients, the applicant should submit the results of Imvigor 130, a phase III randomized study to evaluate the safety and efficacy of atezolizumab monotherapy versus atezolizumab and carboplatin/gemcitabine or cisplatin/gemcitabine in cisplatin-ineligible and -eligible patients.” (Atezolizumab, Tecentriq, 2017) 15. “Further, the single-arm design does not allow to isolate the contribution of the two components of the combination. Of note, based in study KCP-330-001, none of the patients exposed to selinexor in monotherapy obtained a response, only MR and SD were achieved. These data contribute to alleviate the concerns that the effect observed with selinexor in combination with low dose dexamethasone could be mainly driven by the dexamethasone component.” (Selinexor, Nexpovio, 2021)
	Long-term efficacy	Uncertainty concerning the medicine’s long-term efficacy 16. “The median DoR and OS were not evaluable due to the high rate of censoring at the updated analysis (cut-off date11 July 2014), therefore, there is uncertainty on the effect of sonidegib in the long term on DoR and OS.” (Sonidegib, Odomzo, 2015) 17. “The pivotal CLL study (i.e. 312-0116) was terminated early due to efficacy. There are thus no data on long-term efficacy. The magnitude of the treatment effect is therefore not well defined and further follow-up is needed.” (Idelalisib, Zydelig, 2021)
	Relative efficacy	Uncertainty concerning the medicine’s efficacy compared with existing treatments 18. “It is noted that other 5-FU prodrugs, in particular capecitabine, are now widely used fluoropyrimidine analogue as part of triplet regimen for advanced gastric cancer on the basis of efficacy and convenience in clinical practice. On the basis of the submitted data in the MAA Teysuno cannot be considered as an alternative for capecitabine since direct comparison is lacking.” (Tegafur/gimeracil/oteracil, Teysuno, 2011) 19. “Due to the lack of a direct comparison with platinum chemotherapy, the relative efficacy of PARPi compared to platinum chemotherapy has not been defined.” (Talazoparib, Talzenna, 2019)
	Generalisability	Uncertainty concerning the generalisability of the medicine’s efficacy outcomes in the clinical study population to the population specified in the indication 20. “The study population was not a reflection of the patient population regarding performance status, as only a few patients (8.2% in the high dose group) were PS 2 and the rest PS 0-1, and this may lead to selection bias.” (Brigatinib, Alunbrig, 2018) 21. “A further limitation of the studied population is the fact patients with prior treatment with BTK-, bcl-2, and idelalisib were excluded. It is also acknowledged that comparison of the efficacy of duvelisib in CLL after two or more prior treatment to ofatumumab, which was acceptable at the time of the trial initiation, may not be representative in the current context.” (Duvelisib, Copiktra, 2021)
Safety	General safety profile: including, e.g. adverse events, quality of life	Uncertainty concerning the medicine’s general safety profile 22. “The major limitation for safety assessment of KTE-X19 is the limited sample size in the pivotal study ZUMA-2 including 82 subjects, of which only 68 subjects were administered the recommended dose.” (Brexucabtagene autoleucel, Tecartus, 2020) 23. “The main limitations of the safety database are related to difficulties in establishing a causal relationship with avapritinib for key adverse events in the context of non-controlled studies and of a severe underlying condition. In addition, the limited exposure data raise important uncertainties on the actual safety profile of avapritinib.” (Avapritinib, Ayvakyt, 2020) 24. “The main evidence to characterise dostarlimab safety profile comes from ongoing, uncontrolled studies, which is a limitation to contextualise the safety findings.” (Dostarlimab, Jemperli, 2021)
	Subpopulation	Uncertainty concerning the medicine’s safety in a subpopulation 25. “Safety profiles in patients aged ≥75 years old are poorly characterised.” (Lenvatinib, Lenvima, 2015) 26. “Information in special populations such as paediatric patients, pregnant women, in patients with renal or hepatic impairment and in the elderly is currently lacking.” (Allogeneic T cells genetically modified, Zalmoxis, 2016) 27. “The significant limits in the paediatric safety dataset, especially in adolescents, do not allow a full characterisation of the safety profile of entrectinib in this setting.” (Entrectinib, Rozlytrek, 2020)
	Treatment pathway: including, e.g. previous treatment, re-treatment	Uncertainty concerning the positioning of the medicine in the treatment pathway with regard to safety 28. “It is also uncertain whether Tookad VTP adversely influences the ability to later undertake radical therapy, either ‘missed opportunity’ through disease progression or due to the PD effect of vascular occlusion. There is a concern that associated fibrosis may make surgery more difficult and impair post-operative wound healing although the limited available data does not indicate greater difficulty to perform radical prostatectomy.” (Padeliporfin, Tookad, 2017) 29. “The bridging therapies, and more importantly LDC, will lead to AEs that cannot be separated from those resulting from ide-cel infusion.” (Idecabtagene vicleucel, Abecma, 2021)
	Specific adverse events	Uncertainty concerning the occurrence of specific adverse events with the medicine 30. “Tumour progression is inherent in CLL, including transformation/Richter’s syndrome. The incidence (∼10%) of Richter’s syndrome appears somewhat high compared with historical data, but the studies enrolled patients at high risk and specific measures were undertaken to identify cases of transformation. Even though no potential underlying mechanistic explanation has been identified, Richter’s transformation should be considered an important potential risk.” (Venetoclax, Venclyxto, 2016) 31. “A range of immune-related AEs occur at a very low incidence among the All Patients Population. The safety profile of other compounds of the same category indicates that the underlying frequency of these AEs is expected to be low. However, due to the limited number of patients included in the five atezolizumab registration studies, it is considered difficult to draw solid conclusions regarding the exact frequency of the respective immune-related AEs.” (Atezolizumab, Tecentriq, 2017) 32. “Renal lesion findings in non-clinical studies in primate, even though occurring at a significantly higher dose of 3 mg/kg/day than the therapeutic one, are still considered a concern therefore the issue of renal safety needs further clarification.” (Pemigatinib, Pemazyre, 2021)
	Biomarkers	Uncertainty concerning associations between potential biomarkers and the medicine’s safety outcomes 33. “It is still not known whether any biomarkers such as HLA allelic variants are associated with brigatinib-related EOPEs. However, the applicant are endorsing an investigator-initiated study, where it will be explored whether peak reduction in DLCO may be a biomarker for EOPE as well as other relevant secondary endpoints such as systemic inflammatory signatures, immunologic phenotype (e.g. HLA-phenotype), clinical, demographic, and molecular characteristics.” (Brigatinib, Alunbrig, 2018)
	Developmental safety: including, e.g. teratogenicity, embryotoxicity	Uncertainty concerning the medicine’s developmental safety profile 34. “Embryo-foetal toxicity is also an important potential risk that has been described in non-clinical models, however there is no data in humans.” (Avelumab, Bavencio, 2017) 35. “No teratogenic effects were observed in non-clinical studies, however the exposure to fedratinib in these studies was well below the clinical exposure and the relevance of these studies for human safety is therefore limited.” (Fedratinib, inrebic, 2021)
	Combination therapy	Uncertainty concerning the medicine’s safety in combination with another medicine 36. “It is possible that MF patients requiring cytoreductive therapy might in clinical practice be offered symptom reducing ruxolitinib in combination with cytoreductive therapy, such as Hydroxyurea (HU). Combination therapy does not appear to have been studied and the safety of such combination therapy is unknown.” (Ruxolitinib, Jakavi, 2012) 37. “The safety and long-term efficacy in patients with CNS involvement will be followed up in study ML29155, a phase II study of cobimetinib in combination with vemurafenib in active melanoma brain metastases (coBRIM-B) to determine the safety and efficacy of cobimetinib in combination with vemurafenib in patients with active melanoma brain metastases.” (Cobimetinib, Cotellic, 2015)
	Long-term safety	Uncertainty concerning the medicine’s long-term safety 38. “The relatively small safety database of short follow-up of 18 months still represents one of the limitations in the evaluation of the safety related to sonidegib since long-term safety effects (e.g. second primary malignancy induction, cardiac events) may still be underreported.” (Sonidegib, Odomzo, 2015) 39. “For the requested indication, treatment duration is until progression or unacceptable toxicity and therefore long-term exposure might occur, but the safety data for long-term exposure (>12 months, bearing in mind the 95% CI upper bound for median PFS of 13.9 months) are limited.” (Isatuximab, Sarclisa, 2020)
	Relative safety	Uncertainty concerning the medicine’s safety compared to existing treatments 40. “No direct comparison to other taxane therapies has been performed by the applicant.” (Cabazitaxel, Jevtana, 2011) 41. “There is no direct comparison of the sotorasib safety profile with current standard of care therapy (chemotherapy and immunotherapy).” (Sotorasib, Lumykras, 2022)
	Generalisability	Uncertainty concerning the generalisability of the medicine’s safety outcomes in the clinical study population to the population specified in the indication 42. “Safety assessment is based on a single arm study. The patient population enrolled is highly selected: relatively young and fit, yet heavily pre-treated and largely refractory to other treatments. This raises concerns about generalizability of the safety results to real world RRMM patient population.” (Idecabtagene vicleucel, Abecma, 2021)
Pharmacology	Dosing regimen	Uncertainty concerning the optimal dosing regimen of the medicine 43. “In view of the toxicity profile of vandetanib at the 300 mg dose, at least in some patients the safety of the 300 mg dose may be a concern; there is a need to further study the optimal dose with the aim to maximise the benefit-risk balance.” (Vandetanib, Caprelsa, 2012) 44. “The benefit-risk of increasing the gilteritinib dose from 120 mg to 200 mg in patients with lack of response following one treatment cycle is unclear.” (Gilteritinib, Xospata, 2019)
	PK/PD	Uncertainty concerning the medicine’s pharmacokinetics and/or pharmacodynamics 45. “Osimertinib was shown to undergo significant metabolism mediated clearance presumably with the liver as a major site of biotransformation and hence, hepatic impairment might be expected to lead to increased exposure of osimertinib.” (Osimertinib, Tagrisso, 2013) 46. “In light of the claimed site and histology independent indication, the non-clinical pharmacological datapackage is considered insufficient to extrapolate activity in all clinical TRK fusions and tumour histologies including paediatric tumours.” (Entrectinib, Rozlytrek, 2020)
	Potential for interactions: including, e.g. drug–drug interactions and drug–food interactions	Uncertainty concerning the medicine’s potential for interactions 47. “Encorafenib is mainly metabolised via CYP3A4 and there is a possibility of over-exposure due to concomitant use with strong and moderate CYP450 3A4 inhibitors.” (Encorafenib, Braftovi, 2018)
Quality	Product quality	Uncertainty concerning the product quality 48. “Microbial safety of Provenge is ensured by final product release testing, however to improve the overall risk profile of the product prior to its administration, the applicant will develop and implement an additional rapid detection method as an in-process control for microbial quality as detailed in the RMP.” (Sipuleucel-T, Provenge, 2013)
	Manufacturing process	Uncertainty concerning the medicine’s manufacturing process 49. “Although the available data support pooling of results across product and LVV manufacturing process versions, clinical data with JCAR017 manufactured using the LVV in its final commercial version (*n* = 7), for the EU patients, are currently limited.” (Lisocabtagene maraleucel, Breyanzi, 2022)
	Formulation	Uncertainty concerning the formulation of the commercially available product 50. “No patient has been treated with the commercial formulation on the applied dose level.” (Belantamab mafodotin, Blenrep, 2020) 51. “Randomised efficacy results in the target indication are available in a limited and heterogeneous population (*n* = 40 in each arm), with the liquid formulation. Arm G, conducted with the lyo formulation in the pivotal study, is ongoing. Preliminary results in 32 patients were provided at time of response to LoQ, with additional top line results in 42 patients. However, the conduct of a larger (>100 patients) single arm study, using the new formulation, was considered warranted by the CHMP to reach reassurance regarding findings in the pivotal study.” (Polatuzumab vedotin, Polivy, 2020)
Use in clinical practice	Medication errors	Uncertainty concerning the occurrence of medication errors with the medicine 52. “Medication errors have been reported with BLINCYTO. In the pivotal study, the subject incidence of treatment-emergent overdose events was 3.2% (6/189); overdose and accidental overdose (preferred terms) were reported at an incidence of 2.6% (5/189) and 1.1% (2/189), respectively. Medication errors is an important concern related to drug safety (e.g. overdose).” (Blinatumomab, Blincyto, 2015) 53. “The large tablet size may contribute to problems with swallowing.” (Capmatinib, Tabrecta, 2022)
	Manageability of adverse events	Uncertainty concerning the manageability of adverse events caused by the medicine 54. “Based on available data from study 6201, it is currently uncertain what is the best strategy to ensure that diarrhoea is adequately managed, and what is the optimal strategy for anti-diarrhoeal prophylaxis.” (Neratinib, Nerlynx, 2018)

The examples of uncertainties from EPARs are presented as direct quotes. For the three most prevalent subcategories, more quotes are provided.

1L UC, first-line urothelial carcinoma; AE, adverse event; B/R, benefit/risk; Bcl-2, B-cell lymphoma 2; BRAF, b-raf proto-oncogene; BRCA, breast cancer gene; BTK, Bruton’s tyrosine kinase; CHMP, Committee for Medicinal Products for Human Use; CLL, chronic lymphocytic leukaemia; CNS, central nervous system; CRR, complete response rate; CYP, cytochrome enzymes; DLCO, diffusing capacity of the lung for carbon monoxide; DOR, duration of response; EOPEs, early-onset pulmonary events; EPARs, European Public Assessment Reports; EU, European Union; FL, follicular lymphoma; HLA, human leukocyte antigen; Ipd, isatuximab in combination with pomalidomide/dexamethasone; JCAR017, lisocabtagene maraleucel; KTE-X19, brexucabtagene autoleucel; LDC, lymphodepleting chemotherapy; LoQ, list of questions; LVV, lentiviral vector; MA, marketing authorisation; MAA, marketing authorisation application; MF, myelofibrosis; MR, minimal response; ORR, overall response rate; OS, overall survival; PD, pharmacodynamic; PD-L1, programmed death-ligand 1; PD-L2, programmed death-ligand 2; PFS, progression-free survival; PI, proteasome inhibitors; PK/PD, pharmacokinetics/pharmacodynamics; PR, partial response; PS, performance status; RMP, risk management plan; RRMM, relapsed and refractory multiple myeloma; SD, stable disease; TRK, tropomyosin receptor kinase; US, United States; VGPR, very good partial response; VTP, vascular-targeted photodynamic therapy.

### Associations between timing, medicine and regulatory characteristics, and uncertainties

The time of marketing authorisation, medicine characteristics, and regulatory characteristics, and their independent association with number of uncertainties, are shown in Table 3. The median number of uncertainties per medicine increased from 4 (range 0-15) in the period 2011-2014 to 7 (2-23) in the period 2019-2022 (*P* = 0.007). Specifically, efficacy-related uncertainties increased from 1 (0-6) to 3 (1-14) (*P* < 0.001), while the median number of safety-related uncertainties showed no significant difference between 2011 and 2022.

**Table 3:** Associations between characteristics of the cohort and uncertainties

	Medicines cohort	Total uncertainties Median (range)	*P* value	Safety uncertainties Median (range)	*P* value	Efficacy uncertainties Median (range)	*P* value
*n* (%)	121 (100)	800 (100)		404 (51)		322 (40)	
Median	—	6 (0-23)		3 (0-18)		2 (0-14)	
Year of marketing authorisation			**0.007**		0.399		**<0.001**
2011-2014	33 (27)	4 (0-15)		3 (0-12)		1 (0-6)	
2015-2018	42 (35)	5 (1-18)		3 (0-15)		2 (0-8)	
2019-2022	46 (38)	7 (2-23)		3 (0-18)		3 (1-14)	
Medicine characteristics							
Medicine type			0.071		0.165		0.105
Small molecules	73 (60)	6 (0-17)		3 (0-12)		2 (0-8)	
Biologicals	39 (32)	5 (1-12)		2 (0-8)		3 (0-6)	
CGTs	9 (7)	8 (3-23)		3 (1-18)		4 (0-14)	
Therapeutic class (by ATC code)			**0.012**		0.112		0.286
Signal transduction inhibitors	82 (68)	6 (1-17)		3 (0-12)		2 (0-8)	
Tyrosine kinase inhibitors	40 (33)	6 (1-17)		4 (0-12)		2 (0-8)	
Monoclonal antibodies	35 (29)	6 (1-12)		3 (0-8)		3 (0-6)	
Other Signal transduction inhibitors	7 (6)	6 (3-8)		3 (0-7)		2 (1-4)	
CGTs	9 (7)	8 (3-23)		3 (1-18)		4 (0-14)	
Combination therapies	3 (2)	3 (3-6)		2 (1-2)		2 (1-3)	
Other oncology medicines	27 (22)	4 (0-15)		2 (0-9)		2 (0-6)	
First-in-class			0.415		0.075		0.353
Yes	44 (36)	6 (1-18)		3 (0-15)		2 (0-7)	
No	77 (64)	6 (0-23)		3 (0-18)		2 (0-14)	
Regulatory characteristics							
EMA type of marketing authorisation			**0.001**		**0.014**		**0.015**
Standard MA	78 (64)	5 (0-17)		2 (0-12)		2 (0-14)	
Conditional MA	41 (34)	7 (3-23)		4 (0-18)		3 (1-9)	
Exceptional MA	2 (2)	7 (5-8)		3 (2-3)		4 (3-5)	
Expedited and support programmes			0.060		0.084		0.248
Yes	20 (17)	7 (2-23)		4 (0-18)		3 (0-14)	
Accelerated assessment	13 (11)	6 (2-15)		3 (0-8)		2 (0-8)	
PRIME support	9 (7)	10 (3-23)		4 (2-18)		4 (0-14)	
No	101 (83)	6 (0-18)		3 (0-15)		2 (0-7)	
Orphan medicine			0.410		0.292		0.501
Yes	46 (38)	6 (0-23)		3 (0-18)		2 (0-9)	
No	75 (62)	5 (1-17)		3 (0-12)		2 (0-14)	

Therapeutic classes were defined by ATC code. As such, ‘other oncology medicines’ included pyrimidine analogues (L01BC), other hormone antagonists and related agents (L02BX), anti-androgens (L02BB), taxanes (L01CD), poly (ADP ribose) polymerase (PARP) inhibitors (L01XK), cyclin-dependent kinase (CDK) inhibitors (L01EF), anthracyclines and related substances (L01DB) and other antineoplastic agents (L01XX), other immunosuppressants (L04AX), other (HDAC-inhibitors (L01XH), sensitizers used in photodynamic therapy/radiotherapy (L01XD), Hedgehog pathway inhibitors (L01XJ).

The MWU test was carried out on the categories that have an outcome as ‘yes’ or ‘no’ being first-in-class, expedited and support programmes and orphan medicines. The KW test was carried out for categories having more than two characteristics: year of marketing authorisation, medicine type, therapeutic class, and type of marketing authorisation.

ATC, anatomical therapeutic chemical; CGT, cell and gene therapy; CKD, cyclin-dependent kinases; HDAC, histone deacetylase; MA, marketing authorisation; PARP, poly-ADP ribose polymerase; PRIME, priority medicines.

In addition, a higher median number of uncertainties was observed for CGTs [8 (3-23)] compared with other therapeutic classes, i.e. signal transduction inhibitors [6 (1-17)], combination therapies [3 (3-6)], and other oncology medicines [4 (0-15)] (*P* = 0.012). Upon closer examination of all nine CGTs and their uncertainties (*n* = 103, 13%), we observed that uncertainties predominantly evolved around specific adverse events [*n* = 28, 27%; 2 (0-10)], such as carcinogenicity (secondary malignancies), cardiotoxicity, autoimmunity or cytopenia, and effect size [*n* = 19, 18%; 2 (0-5)]. In addition, uncertainties were observed concerning the general safety profile [*n* = 9, 9%; 1, (0-3)], long-term efficacy and safety [both *n* = 5, 10%; 1 (0-1)], and treatment pathway [*n* = 6, 6%; 0 (0-1)]. For this medicine type, uncertainties [*n* = 2, 2%; 0 (0-1)] were also observed regarding the potential differences between the manufacturing process used during clinical development and for the final commercially available product. Of the six quality uncertainties identified in this study, three (50%) were related to CGTs.

Concerning the type of marketing authorisations, most uncertainties were associated with oncology medicines granted a conditional marketing authorisation [7 (3-23)] or those with a marketing authorisation under exceptional circumstances [7 (5-8)] compared with standard marketing authorisation [5 (0-17), *P* = 0.001]. Furthermore, the median number of safety-related uncertainties was higher for medicines granted a conditional marketing authorisation [4 (0-18) versus standard: 2 (0-12); exceptional: 3 (2-3); *P* = 0.014]. The median number of efficacy-related uncertainties was highest for medicines granted marketing authorisation under exceptional circumstances [4 (3-5) versus standard: 2 (0-14); conditional: 3 (1-9); *P* = 0.015]. For orphan-designated medicines, no differences were observed in the number of uncertainties.

**Figure 2:**
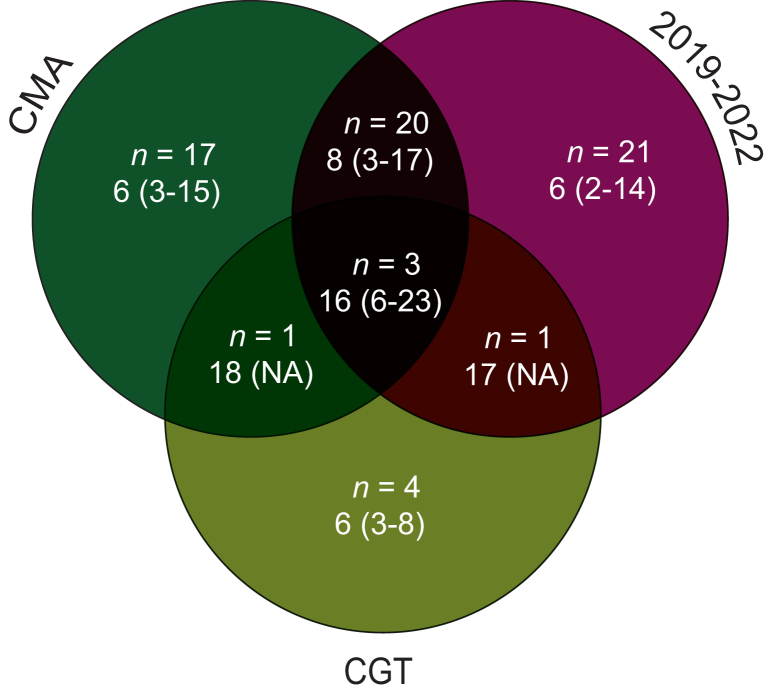
**Timeof authorisation, and medicine and regulatory characteristics related to the median(range)number of uncertainties.** CGT, cell and gene therapy; CMA, conditional marketing authorisation; NA, not applicable.

The linear regression model output indicated that the overlap of two or three characteristics—time of authorisation, type of medicine, and type of marketing authorisation—resulted in a higher number of uncertainties compared with these characteristics separately, which is illustrated in Figure 2 for the actual data in our dataset. Supplementary Table S2, available at https://doi.org/10.1016/j.esmoop.2024.103991, describes these three characteristics for all medicines in our study cohort. Especially CGTs with at least one other characteristic greatly increase the number of uncertainties. Two CGTs had only one other characteristic. The first, lisocabtagene maraleucel (Breyanzi), a CGT granted standard marketing authorisation in 2022, had 17 identified uncertainties. The second, genetically modified allogeneic T cells (Zalmoxis), a CGT granted conditional marketing authorisation in 2016, had 18 identified uncertainties. In addition, three CGTs had all three characteristics: brexucabtagene autoleucel (Tecartus) for the treatment of mantle cell lymphoma (23 uncertainties) and idecabtagene vicleucel (Abecma) and ciltacabtagene autoleucel (Carvykti) for the treatment of multiple myeloma (16 and 6 uncertainties, respectively).

## Discussion

Our research aim was to evaluate the type and number of uncertainties about the benefit-risk balance of oncology medicines as assessed by the EMA at time of initial marketing authorisation in the European Union over the past 12 years. We also aimed to develop a systematic classification of these uncertainties to contribute to improved communication about uncertainties among stakeholders involved in drug development and evaluation, including drug regulatory authorities, pharmaceutical companies, health technology assessment bodies, clinicians, and patients. Among 121 oncology medicines assessed by the EMA, we identified a total of 800 uncertainties, with a median of 6 per medicine. These uncertainties pertained to five different main categories such as safety, efficacy, pharmacology, use in clinical practice, and quality, and 27 subcategories. We found that safety-related uncertainties predominantly concerned specific adverse events or safety in subpopulations, while efficacy-related uncertainties often concerned the effect size and the efficacy in subpopulations. Together, these four subcategories comprised two-thirds of all uncertainties. On the medicine level, we observed an increase in the number of uncertainties per medicine over time, for CGTs, and for medicines granted conditional marketing authorisation.

The number of uncertainties seemed to be especially high for CGTs, with four of the nine CGTs that had a substantially higher number of uncertainties (16-23) compared with other medicines. This higher number of uncertainties may be due to the novel and unique product characteristics, administration methods, and therapeutic outcomes of these therapies. In addition, for medicines that received conditional marketing authorisation more uncertainties were identified compared with standard marketing authorisation. This was expected, as these medicines have been authorised on the basis of non-comprehensive data, necessitating further post-authorisation clinical studies to confirm their benefit-risk profile.^4^^,^^21^ Lastly, for more recently authorised medicines, also an increase in the number of uncertainties was observed. Probably, this is at least partly a consequence of EPAR templates that were updated over time, leading to longer and more detailed benefit-risk discussions. However, the higher number of uncertainties may also be influenced by recent developments such as the authorisation of histology-independent indications. These indications do not target specific cancer types based on their histology or location but target specific genetic mutations or molecular characteristics of cancer cells, regardless of origin.^12^^,^^22^ We observed a higher number of uncertainties at the time of marketing authorisation for two small molecules for histology-independent indications: larotrectinib (Vitrakvi, *n* = 11) and entrectinib (Rozlytrek, *n* = 17). These medicines received conditional marketing authorisation in 2019 and 2020, respectively.

The number of uncertainties did not significantly differ between orphan- and non-orphan-designated medicines. Also, no difference in type of uncertainties (safety, efficacy) was observed. While uncertainties in the evidence can be expected due to the small patient populations, this did not result in more uncertainties being considered for the benefit-risk assessment. One explanation could be that some uncertainties may be more acceptable and/or there is a higher threshold for acknowledging uncertainties for orphan-designated medicines due to inherent limitations. However, the exact reasons remain to be determined.

It is important to recognise that uncertainties are inherent to the development and decision-making processes of innovative medicines. Previous research by Vermeer et al.^23^ has shown that while one-fifth of the safety-related uncertainties identified at the time of initial marketing authorisation and included in EMA risk management plans were resolved within 5 years, new uncertainties emerged over time with a similar rate. These insights emphasise the need for robust post-marketing surveillance, as well as continuous and clear communication about uncertainties throughout the medicine lifecycle. We propose a classification system that could be used as an initial step towards improved communication about these uncertainties. By categorising uncertainties, assessors can more consistently describe specific areas of concern, thereby facilitating communication about and understanding of uncertainties among stakeholders. Moreover, a natural language processing approach may be trained based on our classification system to automate the categorisation of uncertainties for oncology medicines authorised after our study period. Importantly, it is essential to also clearly define the origin of each uncertainty (e.g. the type of data contributing to it, such as data originating from non-randomised studies) as well as the proposed mitigation strategies (e.g. further studies or inclusion of the uncertainty in the medicine's product information). Further research may help to refine these aspects.

Being closest to patients, clinicians are uniquely positioned to address uncertainties concerning treatment outcomes in clinical practice.^24^ By reporting adverse events and clinical experience in scientific literature, and actively contributing real-world data to registries and post-authorisation studies, clinicians can play a significant role in minimising or resolving uncertainties. Our findings may provide a basis to support this effort by improving understanding of the uncertainties that may remain at the time of marketing authorisation.

There are several limitations to our study. Firstly, we did not measure the relative importance or impact of different uncertainties. Secondly, we extracted uncertainties from EPARs rather than the more detailed internal assessment reports. Therefore, although we investigated uncertainties considered ‘key’ for the benefit-risk assessment, this may not result in a complete list of uncertainties. Furthermore, we only focused on initial marketing authorisations for oncology medicines. Different types of uncertainties may be described for other therapeutic areas and stages of development, including pre- and post-authorisation, or stage of the regulatory review. As such, further research is needed to evaluate these uncertainties for medicines with other therapeutic indications and in other stages of drug development.

### Conclusion

In conclusion, our study provides insights into the uncertainties of the benefit-risk balance of oncology medicines that remain at the time of initial marketing authorisation. We observed most uncertainties regarding specific adverse events, effect size, and safety and efficacy in subpopulations. More recently authorised medicines, CGTs and conditionally authorised medicines were associated with most uncertainties. The proposed classification system of uncertainties may help to more consistently describe uncertainties and thereby improve communication among stakeholders, including drug regulatory authorities, pharmaceutical companies, health technology assessment bodies, clinicians, and patients.

## Declaration of generative ai and ai-assisted technologies in the writing process

During the preparation of this work, AI (ChatGPT) was used in order to improve readability. After using this tool, the authors reviewed and edited the content as needed and take full responsibility for the content of the publication.

## Figures and Tables

**Table:** 

Key rules for uncertainty classification During the discussions, strategies for classification were formulated. Here, we present some key rules for clear classification of uncertainties. Based on the header of the benefit-risk balance section (‘uncertainties about the favourable or unfavourable effects’), uncertainties could be initially categorised into efficacy or safety. However, the specifics of the uncertainty (e.g. the meaning) was leading and could overrule that initial classification. For example, an uncertainty text could indicate that there was a safety issue, while in fact the ‘explicit’ issue was related to quality. Next to this, if for example the uncertainty was considered a safety issue, but the uncertainty was described under a favourable effects (efficacy) paragraph of the benefit-risk balance, the uncertainty was still categorised as a safety uncertainty. Regarding safety uncertainties: multiple adverse events in one sentence were categorised as separate safety uncertainties per adverse event. Hierarchy of uncertainties during classification In addition to the key rules, another strategy was applied regarding hierarchy of uncertainties. For instance, if multiple uncertainties were identified within the same text, only one uncertainty was considered most important following a hierarchy. Several combinations of uncertainties were common for which the following hierarchy could be applied. Efficacy a. When uncertainties concerned the ‘full study population’, it was categorised as ‘effect size’. However, if an uncertainty on ‘effect size’ was discussed in a ‘subpopulation’, the uncertainty was categorised as ‘efficacy subpopulation’. The ‘effect size in the subpopulation’ was thus more important than the ‘effect size’ only. Example: “A trend toward ‘PFS benefit’ was observed in the ‘RET negative group’, but results were not statistically significant and therefore the ‘magnitude of clinical benefit’ should be further confirmed.” (Cabozantinib, Cometriq, 2014) b. When uncertainties were either related to ‘effect size’ (mentioning specific endpoint such as OS/PFS) or relative efficacy, these were categorised as ‘effect size’. c. When uncertainties reflected both ‘long-term efficacy’ and related ‘effect size’, these were categorised as ‘long-term efficacy’. d. When uncertainties described both ‘efficacy in a subpopulation’ and ‘long-term efficacy’, these were categorised as ‘efficacy subpopulation’. e. When uncertainties concerned ‘efficacy in a subpopulation after several other treatment options’ were or would be administered, these were categorised as ‘treatment pathway’. Example: “Consequently, the applicant has no data to alleviate the concern that ‘ALKi naive patients’ receiving ceritinib could consequently fail to respond to ‘post-progression therapy with crizotinib or other ALKi’." (Ceritinib, Zykadia, 2015) f. When uncertainties concerned if the clinical study population would reflect the proposed indication population, these were categorised as ‘generalisability’ instead of efficacy subpopulation. The latter were identified as populations that may use the medicine, but may not have been (well) studied due to the characteristics of these populations (age, impairments, disease characteristics). g. When uncertainties about primary endpoints were discussed in combination with the lack of a comparator arm, these were categorised as effect size instead of relative efficacy. The latter concerned lack of comparison to existing treatments. Safety a. When uncertainties reflected ‘specific adverse events’ related to or during ‘long-term safety’, these were categorised as ‘specific adverse events’ as uncertainties regarding general ‘long-term safety’ were categorised ‘long-term safety’. The ‘specific adverse events’ were thus more important than the ‘long-term safety’ only. Example: “Any ‘long-term adverse events’, in particular ‘haematologic long-term toxicity or any organ-manifestations’, have not yet been determined.” (Zanubrutinib, Brukinsa, 2021) b. When uncertainties described how to ‘manage’ specific adverse events, these were categorised as ‘use in clinical practice’. c. When uncertainties concerned the ‘characterisation’ of adverse events, these were categorised as ‘specific adverse events’. Pharmacology a. When uncertainties reflected specific pharmacological pathways, such as PK/PD or interactions, in combination with the description of safety events, these uncertainties were categorised as ‘pharmacology’. Example: “There is uncertainty about the precise ‘exposure–safety relationship’ (with respect to increased AST, ALT, CK, and reduced ALB) although there is a clear increase in ‘frequencies of AEs, including serious events’, in the patients who have escalated to the ‘200 mg dose’, despite small number of events due to limited number of patients that was dose escalated.” (Gilteritinib, Xospata, 2019)

## Appendix 1

Box A1 Rules for the classification of uncertainties
