# Supplementary figures and images for: Uncertainties about the benefit-risk balance of oncology medicines assessed by the European Medicines Agency
Source: ESMO Open. 2024 Dec 9;9(12):103991. doi: 10.1016/j.esmoop.2024.103991 (PMC11696770; doi:10.1016/j.esmoop.2024.103991)

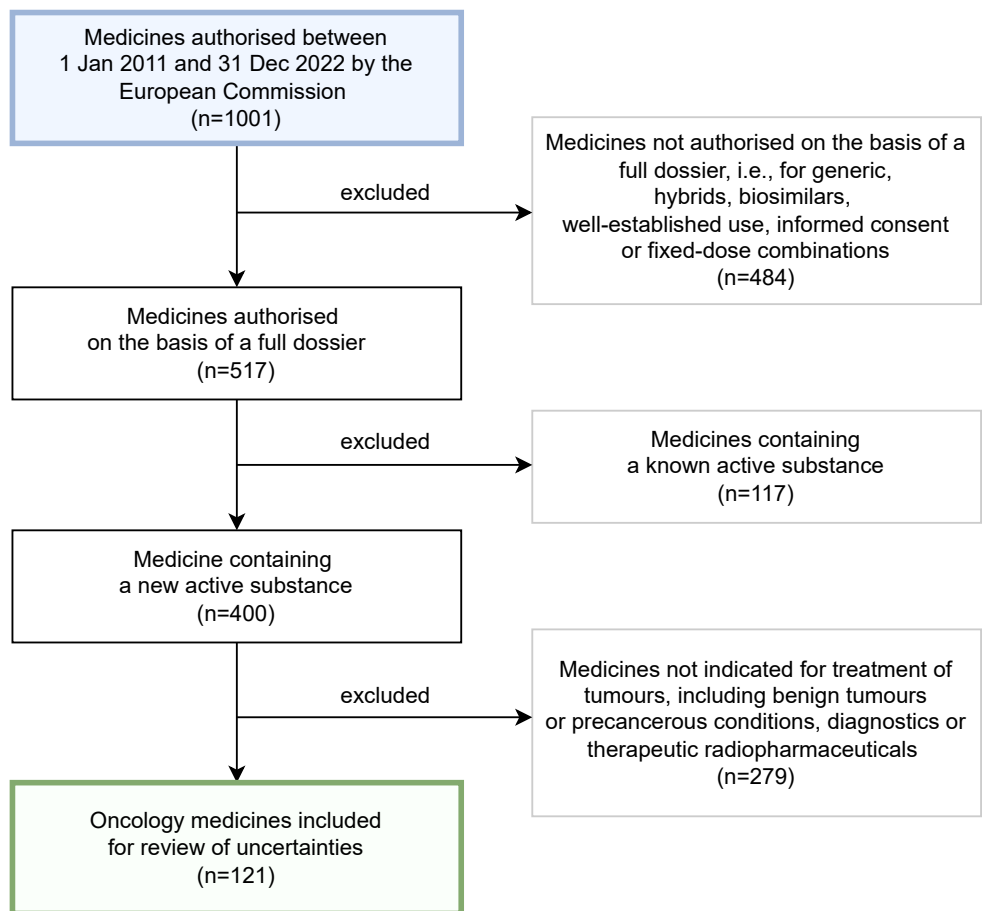

Supplement: Supplementary Figure S1 [file mmc1.pdf]

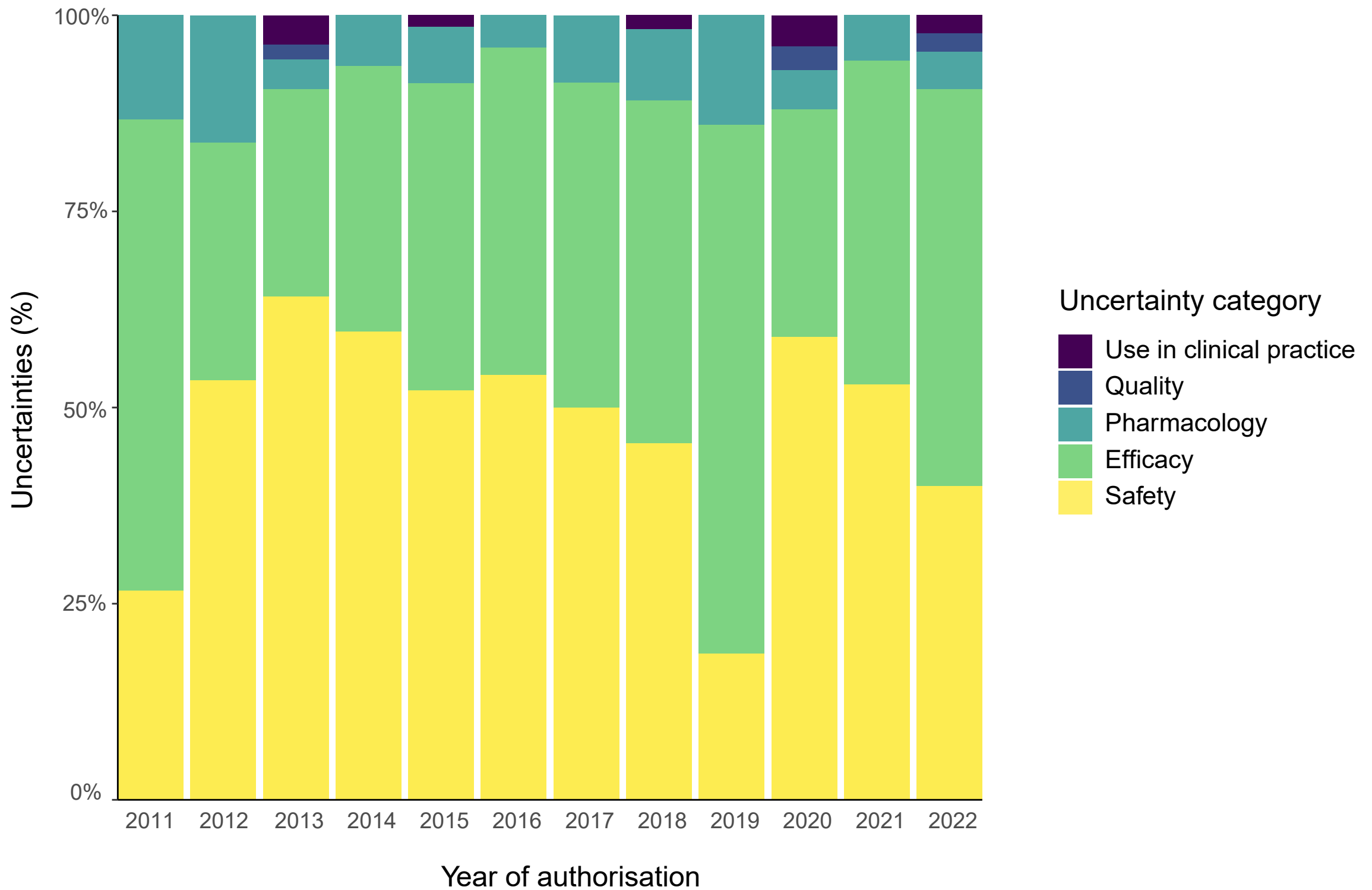

Supplement: Supplementary Figure S2 [file mmc2.pdf]
