# Supplementary material for: Uncertainties about the benefit-risk balance of oncology medicines assessed by the European Medicines Agency
Source: ESMO Open. 2024 Dec 9;9(12):103991. doi: 10.1016/j.esmoop.2024.103991 (PMC11696770; doi:10.1016/j.esmoop.2024.103991)
Supplement: Supplementary Table S1 [file mmc3.pdf]

Table S1. Overview of oncology medicines in our study cohort and their indications at initial marketing authorisation.

Active substance	Brand name	Indication at initial marketing authorisation
**abemaciclib**	Verzenios	Verzenios is indicated for the treatment of women with hormone receptor (HR) positive, human epidermal growth factor receptor 2 (HER2) negative locally advanced or metastatic breast cancer in combination with an aromatase inhibitor or fulvestrant as initial endocrine-based therapy, or in women who have received prior endocrine therapy. In pre- or perimenopausal women, the endocrine therapy should be combined with a luteinising hormone-releasing hormone (LHRH) agonist
**abiraterone**	Zytiga	Zytiga is indicated with prednisone or prednisolone for:<br>1. the treatment of newly diagnosed high risk metastatic hormone sensitive prostate cancer (mHSPC) in adult men in combination with androgen deprivation therapy (ADT) (see section 5.1)<br>2. the treatment of metastatic castration resistant prostate cancer (mCRPC) in adult men who are asymptomatic or mildly symptomatic after failure of androgen deprivation therapy in whom chemotherapy is not yet clinically indicated (see section 5.1)<br>3. the treatment of mCRPC in adult men whose disease has progressed on or after a docetaxel-based chemotherapy regimen.
**acalabrutinib**	Calquence	Calquence as monotherapy or in combination with obinutuzumab is indicated for the treatment of adult patients with previously untreated chronic lymphocytic leukaemia (CLL). Calquence as monotherapy is indicated for the treatment of adult patients with chronic lymphocytic leukaemia (CLL) who have received at least one prior therapy.
**afatinib**	Giotrif	Giotrif as monotherapy is indicated for the treatment of Epidermal Growth Factor Receptor (EGFR) TKInaïve adult patients with locally advanced or metastatic non-small cell lung cancer (NSCLC) with activating EGFR mutation(s)
**aflibercept**	Zaltrap	Zaltrap in combination with irinotecan/5-fluorouracil/folinic acid (FOLFIRI) chemotherapy is<br>indicated in adults with metastatic colorectal cancer (MCRC) that is resistant to or has progressed after an oxaliplatin-containing regimen.
**alectinib**	Alecensa	Alecensa as monotherapy is indicated for the treatment of adult patients with anaplastic lymphoma kinase (ALK)-positive advanced non-small cell lung cancer (NSCLC) previously treated with crizotinib.

Active substance	Brand name	Indication at initial marketing authorisation
**allogeneic T cells genetically modified**	Zalmoxis	Zalmoxis is indicated as adjunctive treatment in haploidentical haematopoietic stem cell transplantation (HSCT) of adult patients with high-risk haematological malignancies
**alpelisib**	Piqray	Piqray is indicated in combination with fulvestrant for the treatment of postmenopausal women, and men, with hormone receptor (HR)-positive, human epidermal growth factor receptor 2 (HER2)-negative, locally advanced or metastatic breast cancer with a PIK3CA mutation after disease progression following endocrine therapy as monotherapy
**amivantamab**	Rybrevant	Rybrevant as monotherapy is indicated for treatment of adult patients with advanced non-small cell lung cancer (NSCLC) with activating epidermal growth factor receptor (EGFR) Exon 20 insertion mutations, after failure of platinum-based therapy
**apalutamide**	Erleada	Erleada is indicated in adult men for the treatment of non-metastatic castration-resistant prostate cancer (NM-CRPC) who are at high risk of developing metastatic disease
**asciminib**	Scemblix	Scemblix is indicated for the treatment of adult patients with Philadelphia chromosome-positive chronic myeloid leukaemia in chronic phase (Ph+ CML-CP) previously treated with two or more tyrosine kinase inhibitors (see section 5.1).
**atezolizumab**	Tecentriq	Tecentriq as monotherapy is indicated for the treatment of adult patients with locally advanced or metastatic urothelial carcinoma (UC) after prior platinum-containing chemotherapy or who are considered cisplatin ineligible (see section 5.1). Tecentriq as monotherapy is indicated for the treatment of adult patients with locally advanced or metastatic non-small cell lung cancer (NSCLC) after prior chemotherapy. Patients with EGFR activating mutations or ALK-positive tumour mutations should also have received targeted therapy before receiving Tecentriq (see section 5.1).
**brexucabtagene autoleucel**	Tecartus	Tecartus is indicated for the treatment of adult patients with relapsed or refractory mantle cell lymphoma (MCL) after two or more lines of systemic therapy including a Bruton’s tyrosine kinase (BTK) inhibitor.
**avapritinib**	Ayvakyt	Ayvakyt is indicated as monotherapy for the treatment of adult patients with unresectable or metastatic gastrointestinal stromal tumours (GIST) harbouring the platelet-derived growth factor receptor alpha (PDGFRA) D842V mutation.
**avelumab**	Bavencio	Bavencio is indicated as monotherapy for the treatment of adult patients with metastatic Merkel cell carcinoma (MCC).

Active substance	Brand name	Indication at initial marketing authorisation
**axicabtagene ciloleucel**	Yescarta	Yescarta is indicated for the treatment of adult patients with relapsed or refractory diffuse large B-cell lymphoma (DLBCL) and primary mediastinal large B-cell lymphoma (PMBCL), after two or more lines of systemic therapy.
**axitinib**	Inlyta	Inlyta is indicated for the treatment of adult patients with advanced renal cell carcinoma (RCC) after failure of prior treatment with sunitinib or a cytokine.
**belantamab mafodotin**	Blenrep	Blenrep is indicated as monotherapy for the treatment of multiple myeloma in adult patients, who have received at least four prior therapies and whose disease is refractory to at least one proteasome inhibitor, one immunomodulatory agent, and an anti-CD38 monoclonal antibody, and who have demonstrated disease progression on the last therapy
**binimetinib**	Mektovi	Binimetinib in combination with encorafenib is indicated for the treatment of adult patients with unresectable or metastatic melanoma with a BRAF V600 mutation
**blinatumomab**	Blincyto	Blincyto is indicated for the treatment of adults with Philadelphia chromosome negative relapsed or refractory B-precursor acute lymphoblastic leukaemia (ALL).
**bosutinib**	Bosulif	Bosulif is indicated for the treatment of adult patients with chronic phase (CP), accelerated phase (AP), and blast phase (BP) Philadelphia chromosome positive chronic myelogenous leukaemia (Ph+<br>CML) previously treated with one or more tyrosine kinase inhibitor(s) and for whom imatinib, nilotinib and dasatinib are not considered appropriate treatment options.
**brentuximab vedotin**	Adcetris	Adcetris is indicated for the treatment of adult patients with relapsed or refractory CD30+ Hodgkin lymphoma (HL):<br>1. following autologous stem cell transplant (ASCT) or<br>2. following at least two prior therapies when ASCT or multi-agent chemotherapy is not a treatment option.<br>Adcetris is indicated for the treatment of adult patients with relapsed or refractory systemic anaplastic large cell lymphoma (sALCL).
**brigatinib**	Alunbrig	Alunbrig is indicated as monotherapy for the treatment of adult patients with anaplastic lymphoma kinase (ALK)-positive advanced non-small cell lung cancer (NSCLC) previously treated with crizotinib.
**cabazitaxel**	Jevtana	Jevtana in combination with prednisone or prednisolone is indicated for the treatment of patients with hormone refractory metastatic prostate

Active substance	Brand name	Indication at initial marketing authorisation
		cancer previously treated with a docetaxel-containing regimen
**cabozantinib**	Cometriq	Cometriq is indicated for the treatment of adult patients with progressive, unresectable locally advanced or metastatic medullary thyroid carcinoma. For patients in whom Rearranged during Transfection (RET) mutation status is not known or is negative, a possible lower benefit should be taken into account before individual treatment decision
**capmatinib**	Tabrecta	Tabrecta as monotherapy is indicated for the treatment of adult patients with advanced non-small cell lung cancer (NSCLC) harbouring alterations leading to mesenchymal-epithelial transition factor gene exon 14 (METex14) skipping, who require systemic therapy following prior treatment with immunotherapy and/or platinum-based chemotherapy.
**carfilzomib**	Kyprolis	Kyprolis in combination with lenalidomide and dexamethasone is indicated for the treatment of adult patients with multiple myeloma who have received at least one prior therapy
**cemiplimab**	Libtayo	Libtayo as monotherapy is indicated for the treatment of adult patients with metastatic or locally advanced cutaneous squamous cell carcinoma who are not candidates for curative surgery or curative radiation
**ceritinib**	Zykadia	Zykadia is indicated for the treatment of adult patients with anaplastic lymphoma kinase (ALK)-positive advanced non-small cell lung cancer (NSCLC) previously treated with crizotinib.
**ciltacabtagene autoleucel**	Carvykti	Carvykti is indicated for the treatment of adult patients with relapsed and refractory multiple myeloma, who have received at least three prior therapies, including an immunomodulatory agent, a proteasome inhibitor and an anti-CD38 antibody and have demonstrated disease progression on the last therapy
**cobimetinib**	Cotellic	Cotellic is indicated for use in combination with vemurafenib for the treatment of adult patients with unresectable or metastatic melanoma with a BRAF V600 mutation
**crizotinib**	Xalkori	Xalkori is indicated for the treatment of adults with previously treated anaplastic lymphoma kinase (ALK)-positive advanced non-small cell lung cancer (NSCLC)
**dabrafenib**	Tafinlar	Dabrafenib is indicated in monotherapy for the treatment of adult patients with unresectable or metastatic melanoma with a BRAF V600 mutation
**dacomitinib**	Vizimpro	Vizimpro, as monotherapy, is indicated for the first-line treatment of adult patients with locally advanced or metastatic non-small cell lung cancer

Active substance	Brand name	Indication at initial marketing authorisation
		(NSCLC) with epidermal growth factor receptor (EGFR)-activating mutations.
**daratumumab**	Darzalex	Darzalex as monotherapy is indicated for the treatment of adult patients with relapsed and refractory multiple myeloma, whose prior therapy included a proteasome inhibitor and an immunomodulatory agent and who have demonstrated disease progression on the last therapy
**darolutamide**	Nubeqa	Nubeqa is indicated for the treatment of adult men with non-metastatic castration resistant prostate cancer (nmCRPC) who are at high risk of developing metastatic disease
**decitabine**	Dacogen	Dacogen is indicated for the treatment of adult patients aged 65 years and above with newly diagnosed<br>de novo or secondary acute myeloid leukaemia (AML), according to the World Health Organisation<br>(WHO) classification, who are not candidates for standard induction chemotherapy.
**dinutuximab**	Unituxin	Unituxin is indicated for the treatment of high-risk neuroblastoma in patients aged 12 months to 17 years, who have previously received induction chemotherapy and achieved at least a partial response, followed by myeloablative therapy and autologous stem cell transplantation (ASCT). It is administered in combination with granulocyte-macrophage colony-stimulating factor (GM-CSF), interleukin-2 (IL-2), and isotretinoin.
**dostarlimab**	Jemperli	Jemperli is indicated as monotherapy for the treatment of adult patients with mismatch repair deficient (dMMR)/microsatellite instability-high (MSI-H) recurrent or advanced endometrial cancer (EC) that has progressed on or following prior treatment with a platinum-containing regimen
**durvalumab**	Imfinzi	Imfinzi as monotherapy is indicated for the treatment of locally advanced, unresectable non-small cell lung cancer (NSCLC) in adults whose tumours express PD-L1 on ≥ 1% of tumour cells and whose disease has not progressed following platinum-based chemoradiation therapy
**duvelisib**	Copiktra	Copiktra monotherapy is indicated for the treatment of adult patients with: • Relapsed or refractory chronic lymphocytic leukaemia (CLL) after at least two prior therapies. (see section 4.4.and 5.1). • Follicular lymphoma (FL) that is refractory to at least two prior systemic therapies. (see section 4.4.and 5.1).
**elotuzumab**	Empliciti	Empliciti is indicated in combination with lenalidomide and dexamethasone for the treatment of multiple myeloma in adult patients who have received at least one prior therapy

Active substance	Brand name	Indication at initial marketing authorisation
**encorafenib**	Braftovi	Encorafenib in combination with binimetinib is indicated for the treatment of adult patients with unresectable or metastatic melanoma with a BRAF V600 mutation
**enfortumab vedotin**	Padcev	Padcev as monotherapy is indicated for the treatment of adult patients with locally advanced or metastatic urothelial cancer who have previously received a platinum-containing chemotherapy and a programmed death receptor-1 or programmed death-ligand 1 inhibitor (see section 5.1).
**entrectinib**	Rozlytrek	Rozlytrek as monotherapy is indicated for the treatment of adult and paediatric patients 12 years of age and older with solid tumours expressing a neurotrophic tyrosine receptor kinase (NTRK) gene fusion, • who have a disease that is locally advanced, metastatic or where surgical resection is likely to result in severe morbidity, and • who have not received a prior NTRK inhibitor 3 • who have no satisfactory treatment options (see sections 4.4 and 5.1). Rozlytrek as monotherapy is indicated for the treatment of adult patients with ROS1-positive, advanced non-small cell lung cancer (NSCLC) not previously treated with ROS1 inhibitors.
**enzalutamide**	Xtandi	Xtandi is indicated for the treatment of adult men with metastatic castration-resistant prostate cancer whose disease has progressed on or after docetaxel therapy.
**eribulin**	Halaven	Halaven monotherapy is indicated for the treatment of patients with locally advanced or metastatic breast cancer who have progressed after at least two chemotherapeutic regimens for advanced disease (see section 5.1). Prior therapy should have included an anthracycline and a taxane unless patients were not suitable for these treatments.
**fedratinib**	Inrebic	Inrebic is indicated for the treatment of disease-related splenomegaly or symptoms in adult patients with primary myelofibrosis, post polycythaemia vera myelofibrosis or post essential thrombocythaemia myelofibrosis who are Janus Associated Kinase (JAK) inhibitor naïve or have been treated with ruxolitinib.
**gemtuzumab ozogamicin**	Mylotarg	Mylotarg is indicated for combination therapy with daunorubicin (DNR) and cytarabine (AraC) for the treatment of patients age 15 years and above with previously untreated, de novo CD33-positive acute myeloid leukaemia (AML), except acute promyelocytic leukaemia (APL)
**gilteritinib**	Xospata	Xospata is indicated as monotherapy for the treatment of adult patients who have relapsed or refractory acute myeloid leukaemia (AML) with a FLT3 mutation
**glasdegib**	Daurismo	Daurismo is indicated, in combination with low-dose cytarabine, for the treatment of newly

Active substance	Brand name	Indication at initial marketing authorisation
		diagnosed de novo or secondary acute myeloid leukaemia (AML) in adult patients who are not candidates for standard induction chemotherapy.
**ibrutinib**	Imbruvica	Imbruvica is indicated for the treatment of adult patients with relapsed or refractory mantle cell lymphoma (MCL). Imbruvica is indicated for the treatment of adult patients with chronic lymphocytic leukaemia (CLL) who have received at least one prior therapy, or in first line in the presence of 17p deletion or TP53 mutation in patients unsuitable for chemo-immunotherapy
**idecabtagene vicleucel**	Abecma	Abecma is indicated for the treatment of adult patients with relapsed and refractory multiple myeloma who have received at least three prior therapies, including an immunomodulatory agent, a proteasome inhibitor and an anti-CD38 antibody and have demonstrated disease progression on the last therapy.
**idelalisib**	Zydelig	Zydelig is indicated in combination with rituximab for the treatment of adult patients with chronic lymphocytic leukaemia (CLL): • who have received at least one prior therapy, or • as first line treatment in the presence of 17p deletion or TP53 mutation in patients unsuitable for chemo-immunotherapy. Zydelig is indicated as monotherapy for the treatment of adult patients with follicular lymphoma (FL) that is refractory to two prior lines of treatment.
**inotuzumab ozogamicin**	Besponsa	Besponsa is indicated as monotherapy for the treatment of adults with relapsed or refractory CD22- positive B cell precursor acute lymphoblastic leukaemia (ALL). Adult patients with Philadelphia chromosome positive (Ph+) relapsed or refractory B cell precursor ALL should have failed treatment with at least 1 tyrosine kinase inhibitor (TKI).
**ipilimumab**	Yervoy	Yervoy is indicated for the treatment of advanced (unresectable or metastatic) melanoma in adults who have received prior therapy.
**isatuximab**	Sarclisa	Sarclisa is indicated, in combination with pomalidomide and dexamethasone, for the treatment of adult patients with relapsed and refractory multiple myeloma (MM) who have received at least two prior therapies including lenalidomide and a proteasome inhibitor (PI) and have demonstrated disease progression on the last therapy.
**ixazomib**	Ninlaro	Ninlaro in combination with lenalidomide and dexamethasone is indicated for the treatment of adult patients with multiple myeloma who have received at least one prior therapy.
**larotrectinib**	Vitrakvi	Viktrakvi as monotherapy is indicated for the treatment of adult and paediatric patients with solid

Active substance	Brand name	Indication at initial marketing authorisation
		tumours that display a Neurotrophic Tyrosine Receptor Kinase (NTRK) gene fusion, - who have a disease that is locally advanced, metastatic or where surgical resection is likely to result in severe morbidity, and - who have no satisfactory treatment options
**lenvatinib**	Lenvima	Lenvima is indicated for the treatment of adult patients with progressive, locally advanced or metastatic differentiated (papillary/follicular/Hürhle cell) thyroid carcinoma (DTC), refractory to radioactive iodine (RAI).
**lisocabtagene maraleucel**	Breyanzi	Breyanzi is indicated for the treatment of adult patients with relapsed or refractory diffuse large B-cell lymphoma (DLBCL), primary mediastinal large B-cell lymphoma (PMBCL) and follicular lymphoma grade 3B (FL3B), after two or more lines of systemic therapy.
**loncastuximab tesirine**	Zynlonta	Zynlonta as monotherapy is indicated for the treatment of adult patients with relapsed or refractory diffuse large B-cell lymphoma (DLBCL) and high-grade B-cell lymphoma (HGBL), after two or more lines of systemic therapy.
**lorlatinib**	Lorviqua	Lorviqua as monotherapy is indicated for the treatment of adult patients with anaplastic lymphoma kinase (ALK)-positive advanced non-small cell lung cancer (NSCLC) whose disease has progressed after: • alectinib or ceritinib as the first ALK tyrosine kinase inhibitor (TKI) therapy; or • crizotinib and at least one other ALK TKI.
**midostaurin**	Rydapt	Rydapt is indicated: · in combination with standard daunorubicin and cytarabine induction and high-dose cytarabine consolidation chemotherapy, and for patients in complete response followed by Rydapt single agent maintenance therapy, for adult patients with newly diagnosed acute myeloid leukaemia (AML) who are FLT3 mutation-positive (see section 4.2); · as monotherapy for the treatment of adult patients with aggressive systemic mastocytosis (ASM), systemic mastocytosis with associated haematological neoplasm (SM-AHN), or mast cell leukaemia (MCL).
**mogamulizumab**	Poteligeo	Poteligeo is indicated for the treatment of adult patients with mycosis fungoides (MF) or Sézary syndrome (SS) who have received at least one prior systemic therapy
**mosunetuzumab**	Lunsumio	Lunsumio as monotherapy is indicated for the treatment of adult patients with relapsed or refractory follicular lymphoma (FL) who have received at least two prior systemic therapies.
**moxetumomab pasudotox**	Lumoxiti	Lumoxiti as monotherapy is indicated for the treatment of adult patients with relapsed or refractory hairy cell leukaemia (HCL) after receiving at least two prior systemic therapies,

Active substance	Brand name	Indication at initial marketing authorisation
		including treatment with a purine nucleoside analogue (PNA).
**necitumumab**	Portrazza	Portrazza in combination with gemcitabine and cisplatin chemotherapy is indicated for the treatment of adult patients with locally advanced or metastatic epidermal growth factor receptor (EGFR) expressing squamous non-small cell lung cancer who have not received prior chemotherapy for this condition.
**neratinib**	Nerlynx	Nerlynx is indicated for the extended adjuvant treatment of adult patients with early-stage hormone receptor positive HER2-overexpressed/amplified breast cancer and who are less than one year from the completion of prior adjuvant trastuzumab based therapy.
**nintedanib**	Vargatef	Vargatef is indicated in combination with docetaxel for the treatment of adult patients with locally advanced, metastatic or locally recurrent non-small cell lung cancer (NSCLC) of adenocarcinoma tumour histology after first-line chemotherapy.
**niraparib**	Zejula	Zejula is indicated as monotherapy for the maintenance treatment of adult patients with platinum-sensitive relapsed high grade serous epithelial ovarian, fallopian tube, or primary peritoneal cancer who are in response (complete or partial) to platinum-based chemotherapy.
**nivolumab**	Opdivo	Opdivo as monotherapy is indicated for the treatment of advanced (unresectable or metastatic) melanoma in adults
**nivolumab**	Nivolumab BMS	Nivolumab BMS is indicated for the treatment of locally advanced or metastatic squamous non-small cell lung cancer (NSCLC) after prior chemotherapy in adults
**nivolumab/relatlimab**	Opdualag	Opdualag is indicated for the first-line treatment of advanced (unresectable or metastatic) melanoma in adults and adolescents 12 years of age and older with tumour cell PD-L1 expression < 1%.
**obinutuzumab**	Gazyvaro	Gazyvaro in combination with chlorambucil is indicated for the treatment of adult patients with previously untreated chronic lymphocytic leukaemia (CLL) and with comorbidities making them unsuitable for full-dose fludarabine based therapy
**olaparib**	Lynparza	Lynparza is indicated as monotherapy for the maintenance treatment of adult patients with platinum-sensitive relapsed BRCA-mutated (germline and/or somatic) high grade serous epithelial ovarian, fallopian tube, or primary peritoneal cancer who are in response (complete response or partial response) to platinum-based chemotherapy.
**olaratumab**	Lartruvo	Lartruvo is indicated in combination with doxorubicin for the treatment of adult patients with

Active substance	Brand name	Indication at initial marketing authorisation
		advanced soft tissue sarcoma who are not amenable to curative treatment with surgery or radiotherapy and who have not been previously treated with doxorubicin
**osimertinib**	Tagrisso	Tagrisso is indicated for the treatment of adult patients with locally advanced or metastatic epidermal growth factor receptor (EGFR) T790M mutation-positive non-small-cell lung cancer (NSCLC).
**padeliporfin**	Tookad	Tookad is indicated as monotherapy for adult patients with previously untreated, unilateral, low-risk, adenocarcinoma of the prostate with a life expectancy ≥ 10 years and: - Clinical stage T1c or T2a, - Gleason Score ≤ 6, based on high-resolution biopsy strategies, - PSA ≤ 10 ng/mL, - 3 positive cancer cores with a maximum cancer core length of 5 mm in any one core or 1-2 positive cancer cores with ≥ 50 % cancer involvement in any one core or a PSA density ≥ 0.15 ng/mL/cm3 .
**palbociclib**	Ibrance	Ibrance is indicated for the treatment of hormone receptor (HR)-positive, human epidermal growth factor receptor 2 (HER2)-negative locally advanced or metastatic breast cancer: - in combination with an aromatase inhibitor; - in combination with fulvestrant in women who have received prior endocrine therapy (see section 5.1). In pre- or perimenopausal women, the endocrine therapy should be combined with a luteinizing hormone-releasing hormone (LHRH) agonist.
**panobinostat**	Farydak	Farydak, in combination with bortezomib and dexamethasone, is indicated for the treatment of adult patients with relapsed and/or refractory multiple myeloma who have received at least two prior regimens including bortezomib and an immunomodulatory agent.
**pembrolizumab**	Keytruda	Keytruda as monotherapy is indicated for the treatment of advanced (unresectable or metastatic) melanoma in adults.
**pemigatinib**	Pemazyre	Pemazyre monotherapy is indicated for the treatment of adults with locally advanced or metastatic cholangiocarcinoma with a fibroblast growth factor receptor 2 (FGFR2) fusion or rearrangement that have progressed after at least one prior line of systemic therap
**pertuzumab**	Perjeta	Perjeta is indicated for use in combination with trastuzumab and docetaxel in adult patients with HER2-<br>positive metastatic or locally recurrent unresectable breast cancer, who have not received previous antiHER2 therapy or chemotherapy for their metastatic disease.
**pixantrone**	Pixuvri	Pixuvri is indicated as monotherapy for the treatment of adult patients with multiply relapsed or

Active substance	Brand name	Indication at initial marketing authorisation
		refractory aggressive Non-Hodgkin B-cell Lymphomas (NHL). The benefit of pixantrone treatment has not been established in patients when used as fifth line or greater chemotherapy in patients who are refractory to last therapy.
**polatuzumab vedotin**	Polivy	Polivy in combination with bendamustine and rituximab is indicated for the treatment of adult patients with relapsed/refractory diffuse large B-cell lymphoma (DLBCL) who are not candidates for haematopoietic stem cell transplant.
**pomalidomide**	Imnovid	Pomalidomide Celgene in combination with dexamethasone is indicated in the treatment of adult patients with relapsed and refractory multiple myeloma who have received at least two prior treatment regimens, including both lenalidomide and bortezomib, and have demonstrated disease progression on the last therapy.
**ponatinib**	Iclusig	Iclusig is indicated in adult patients with · chronic phase, accelerated phase, or blast phase chronic myeloid leukaemia (CML) who are resistant to dasatinib or nilotinib; who are intolerant to dasatinib or nilotinib and for whom subsequent treatment with imatinib is not clinically appropriate; or who have the T315I mutation · Philadelphia chromosome positive acute lymphoblastic leukaemia (Ph+ ALL) who are resistant to dasatinib; who are intolerant to dasatinib and for whom subsequent treatment with imatinib is not clinically appropriate; or who have the T315I mutation.
**pralsetinib**	Gavreto	Gavreto is indicated as monotherapy for the treatment of adult patients with rearranged during transfection (RET) fusion-positive advanced non-small cell lung cancer (NSCLC) not previously treated with a RET inhibitor
**ramucirumab**	Cyramza	Cyramza in combination with paclitaxel is indicated for the treatment of adult patients with advanced gastric cancer or gastro-oesophageal junction adenocarcinoma with disease progression after prior platinum and fluoropyrimidine chemotherapy (see section 5.1). Cyramza monotherapy is indicated for the treatment of adult patients with advanced gastric cancer or gastro-oesophageal junction adenocarcinoma with disease progression after prior platinum or fluoropyrimidine chemotherapy, for whom treatment in combination with paclitaxel is not appropriate
**regorafenib**	Stivarga	Stivarga is indicated for the treatment of adult patients with metastatic colorectal cancer (CRC) who have been previously treated with, or are not

Active substance	Brand name	Indication at initial marketing authorisation
		considered candidates for, available therapies. These include fluoropyrimidine-based chemotherapy, an anti-VEGF therapy and an anti-EGFR therapy
**ribociclib**	Kisqali	Kisqali in combination with an aromatase inhibitor is indicated for the treatment of postmenopausal women with hormone receptor (HR)-positive, human epidermal growth factor receptor 2 (HER2)-negative locally advanced or metastatic breast cancer as initial endocrine-based therapy.
**ripretinib**	Qinlock	Qinlock is indicated for the treatment of adult patients with advanced gastrointestinal stromal tumour (GIST) who have received prior treatment with three or more kinase inhibitors, including imatinib.
**rucaparib**	Rubraca	Rubraca is indicated as monotherapy treatment of adult patients with platinum sensitive, relapsed or progressive, BRCA mutated (germline and/or somatic), high-grade epithelial ovarian, fallopian tube, or primary peritoneal cancer, who have been treated with two or more prior lines of platinum based chemotherapy, and who are unable to tolerate further platinum based chemotherapy.
**ruxolitinib**	Jakavi	Jakavi is indicated for the treatment of disease-related splenomegaly or symptoms in adult patients with primary myelofibrosis (also known as chronic idiopathic myelofibrosis), post polycythaemia vera myelofibrosis or post essential thrombocythaemia myelofibrosis
**sacituzumab govitecan**	Trodelvy	Trodelvy as monotherapy is indicated for the treatment of adult patients with unresectable or metastatic triple-negative breast cancer (mTNBC) who have received two or more prior systemic therapies, including at least one of them for advanced disease
**selinexor**	Nexpovio	Nexpovio is indicated in combination with dexamethasone for the treatment of multiple myeloma in adult patients who have received at least four prior therapies and whose disease is refractory to at least two proteasome inhibitors, two immunomodulatory agents and an anti-CD38 monoclonal antibody, and who have demonstrated disease progression on the last therapy.
**selpercatinib**	Retsevmo	Retsevmo as monotherapy is indicated for the treatment of adults with: – advanced RET fusion-positive non-small cell lung cancer (NSCLC) who require systemic therapy following prior treatment with immunotherapy and/or platinum-based chemotherapy – advanced RET fusion-positive thyroid cancer who require systemic therapy following prior treatment with sorafenib and/or lenvatinib Retsevmo as monotherapy is indicated for the treatment of adults and adolescents 12 years

Active substance	Brand name	Indication at initial marketing authorisation
		and older with advanced RET-mutant medullary thyroid cancer (MTC) who require systemic therapy following prior treatment with cabozantinib and/or vandetanib.
**sipuleucel-T**	Provenge	Provenge is indicated for treatment of asymptomatic or minimally symptomatic metastatic (nonvisceral) castrate resistant prostate cancer in male adults in whom chemotherapy is not yet clinically indicated.
**sonidegib**	Odomzo	Odomzo is indicated for the treatment of adult patients with locally advanced basal cell carcinoma (BCC) who are not amenable to curative surgery or radiation therapy.
**sotorasib**	Lumykras	Lumykras as monotherapy is indicated for the treatment of adults with advanced non-small cell lung cancer (NSCLC) with KRAS G12C mutation and who have progressed after at least one prior line of systemic therapy.
**tafasitamab**	Minjuvi	Minjuvi is indicated in combination with lenalidomide followed by Minjuvi monotherapy for the treatment of adult patients with relapsed or refractory diffuse large B-cell lymphoma (DLBCL) who are not eligible for autologous stem cell transplant (ASCT)
**tagraxofusp**	Elzonris	Elzonris is indicated as monotherapy for the first-line treatment of adult patients with blastic plasmacytoid dendritic cell neoplasm (BPDCN)
**talazoparib**	Talzenna	Talzenna is indicated as monotherapy for the treatment of adult patients with germline BRCA1/2-mutations, who have HER2-negative locally advanced or metastatic breast cancer. Patients should have been previously treated with an anthracycline and/or a taxane in the (neo)adjuvant, locally advanced or metastatic setting unless patients were not suitable for these treatments (see section 5.1). Patients with hormone receptor (HR)-positive breast cancer should have been treated with a prior endocrine-based therapy, or be considered unsuitable for endocrine-based therapy.
**talimogene laherparepvec**	Imlygic	Imlygic is indicated for the treatment of adults with unresectable melanoma that is regionally or distantly metastatic (Stage IIIB, IIIC and IVM1a) with no bone, brain, lung or other visceral disease
**tebentafusp**	Kimmtrak	Kimmtrak is indicated as monotherapy for the treatment of human leukocyte antigen (HLA)-A*02:01-positive adult patients with unresectable or metastatic uveal melanoma.
**teclistamab**	Tecvayli	Tecvayli is indicated as monotherapy for the treatment of adult patients with relapsed and refractory multiple myeloma, who have received at least three prior therapies, including an immunomodulatory agent, a proteasome inhibitor,

Active substance	Brand name	Indication at initial marketing authorisation
		and an anti-CD38 antibody and have demonstrated disease progression on the last therapy.
**tegafur/gimeracil/ oteracil**	Teysuno	Teysuno is indicated in adults for the treatment of advanced gastric cancer when given in combination with cisplatin
**tepotinib**	Tepmetko	Tepmetko as monotherapy is indicated for the treatment of adult patients with advanced non-small cell lung cancer (NSCLC) harbouring alterations leading to mesenchymal-epithelial transition factor gene exon 14 (METex14) skipping, who require systemic therapy following prior treatment with immunotherapy and/or platinum-based chemotherapy.
**tisagenlecleucel**	Kymriah	Kymriah is indicated for the treatment of: · Paediatric and young adult patients up to 25 years of age with B-cell acute lymphoblastic leukaemia (ALL) that is refractory, in relapse post-transplant or in second or later relapse. · Adult patients with relapsed or refractory diffuse large B-cell lymphoma (DLBCL) after two or more lines of systemic therapy.
**tivozanib**	Fotivda	Fotivda is indicated for the first line treatment of adult patients with advanced renal cell carcinoma (RCC) and for adult patients who are VEGFR and mTOR pathway inhibitor-naïve following disease progression after one prior treatment with cytokine therapy for advanced RCC
**trametinib**	Mekinist	Trametinib is indicated for the treatment of adult patients with unresectable or metastatic melanoma with a BRAF V600 mutation. Trametinib has not demonstrated clinical activity in patients who have progressed on a prior BRAF inhibitor therapy
**trastuzumab deruxtecan**	Enhertu	Enhertu as monotherapy is indicated for the treatment of adult patients with unresectable or metastatic HER2-positive breast cancer who have received two or more prior anti-HER2-based regimens.
**trastuzumab emtansine**	Kadcyla	Kadcyla, as a single agent, is indicated for the treatment of adult patients with HER2-positive, unresectable locally advanced or metastatic breast cancer who previously received trastuzumab and a taxane, separately or in combination. Patients should have either: • Received prior therapy for locally advanced or metastatic disease, or • Developed disease recurrence during or within six months of completing adjuvant therapy.
**trifluridine / tipiracil**	Lonsurf	Lonsurf is indicated for the treatment of adult patients with metastatic colorectal cancer (CRC) who have been previously treated with, or are not considered candidates for, available therapies including fluoropyrimidine-, oxaliplatin- and irinotecan-based chemotherapies, anti-VEGF agents, and anti-EGFR agents.

Active substance	Brand name	Indication at initial marketing authorisation
**tucatinib**	Tukysa	Tukysa is indicated in combination with trastuzumab and capecitabine for the treatment of adult patients with HER2-positive locally advanced or metastatic breast cancer who have received at least 2 prior anti-HER2 treatment regimens.
**vandetanib**	Caprelsa	Caprelsa is indicated for the treatment of aggressive and symptomatic medullary thyroid cancer (MTC) in patients with unresectable locally advanced or metastatic disease.<br>For patients in whom Rearranged during Transfection (RET) mutation is not known or is negative, a<br>possible lower benefit should be taken into account before individual treatment decision
**vemurafenib**	Zelboraf	Vemurafenib is indicated in monotherapy for the treatment of adult patients with BRAF V600 mutation-positive unresectable or metastatic melanoma
**venetoclax**	Venclyxto	Venclyxto monotherapy is indicated for the treatment of chronic lymphocytic leukaemia (CLL) in the presence of 17p deletion or TP53 mutation in adult patients who are unsuitable for or have failed a B-cell receptor pathway inhibitor. Venclyxto monotherapy is indicated for the treatment of CLL in the absence of 17p deletion or TP53 mutation in adult patients who have failed both chemoimmunotherapy and a B-cell receptor pathway inhibitor
**vismodegib**	Erivedge	Erivedge is indicated for the treatment of adult patients with:<br>• symptomatic metastatic basal cell carcinoma<br>• locally advanced basal cell carcinoma inappropriate for surgery or radiotherapy
**zanubrutinib**	Brukinsa	Brukinsa as monotherapy is indicated for the treatment of adult patients with Waldenström’s macroglobulinaemia (WM) who have received at least one prior therapy, or in first line treatment for patients unsuitable for chemo-immunotherapy.
