# Supplementary material for: Uncertainties about the benefit-risk balance of oncology medicines assessed by the European Medicines Agency
Source: ESMO Open. 2024 Dec 9;9(12):103991. doi: 10.1016/j.esmoop.2024.103991 (PMC11696770; doi:10.1016/j.esmoop.2024.103991)
Supplement: Supplementary Table S2 [file mmc4.pdf]

Table S2. Overview of time, medicine and regulatory characteristics per medicine

Active substance	Brand name	Date of MA by EC	Medicine type	Type of authorisation
**abemaciclib**	Verzenios	27-9-2018	Small molecule	SMA
**abiraterone**	Zytiga	5-9-2011	Small molecule	SMA
**acalabrutinib**	Calquence	5-11-2020	Small molecule	SMA
**afatinib**	Giotrif	25-9-2013	Small molecule	SMA
**aflibercept**	Zaltrap	1-2-2013	Biological	SMA
**alectinib**	Alecensa	16-2-2017	Small molecule	CMA
**allogeneic T cells genetically modified**[a]	Zalmoxis	18-8-2016	CGT	CMA
**alpelisib**	Piqray	27-7-2020	Small molecule	SMA
**amivantamab**	Rybrevant	9-12-2021	Biological	CMA
**apalutamide**	Erleada	14-1-2019	Small molecule	SMA
**asciminib**	Scemblix	25-8-2022	Small molecule	SMA
**atezolizumab**	Tecentriq	21-9-2017	Biological	SMA
**brexucabtagene autoleucel**	Tecartus	14-12-2020	CGT	CMA
**sipuleucel-T**	Provenge	6-9-2013	CGT	SMA
**avapritinib**	Ayvakyt	24-9-2020	Biological	CMA
**avelumab**	Bavencio	18-9-2017	Biological	CMA
**axicabtagene ciloleucel**	Yescarta	23-8-2018	CGT	SMA
**axitinib**	Inlyta	3-9-2012	Biological	SMA
**belantamab mafodotin**	Blenrep	25-8-2020	Small molecule	CMA
**binimetinib**	Mektovi	20-9-2018	Biological	SMA
**blinatumomab**	Blincyto	23-11-2015	Small molecule	CMA
**bosutinib**	Bosulif	27-3-2013	Biological	CMA
**brentuximab vedotin**	Adcetris	25-10-2012	Small molecule	CMA
**brigatinib**	Alunbrig	22-11-2018	Small molecule	SMA
**cabazitaxel**	Jevtana	17-3-2011	Small molecule	SMA
**cabozantinib**	Cometriq	21-3-2014	Small molecule	CMA
**capmatinib**	Tabrecta	20-6-2022	Small molecule	SMA
**carfilzomib**	Kyprolis	19-11-2015	Biological	SMA
**cemiplimab**	Libtayo	28-6-2019	Small molecule	CMA
**ceritinib**	Zykadia	6-5-2015	Small molecule	CMA
**ciltacabtagene autoleucel**	Carvykti	25-5-2022	CGT	CMA
**cobimetinib**	Cotellic	20-11-2015	Small molecule	SMA
**crizotinib**	Xalkori	23-10-2012	Small molecule	CMA
**dabrafenib**	Tafinlar	26-8-2013	Small molecule	SMA
**dacomitinib**	Vizimpro	2-4-2019	Biological	SMA
**daratumumab**	Darzalex	20-5-2016	Small molecule	CMA

**darolutamide**	Nubeqa	27-3-2020	Small molecule	SMA
**decitabine**	Dacogen	20-9-2012	Biological	SMA
**dinutuximab**	Unituxin	14-8-2015	Biological	SMA
**dostarlimab**	Jemperli	21-4-2021	Biological	CMA
**durvalumab**	Imfinzi	21-9-2018	Small molecule	SMA
**duvelisib**	Copiktra	19-5-2021	Biological	SMA
**elotuzumab**	Empliciti	11-5-2016	Small molecule	SMA
**encorafenib**	Braftovi	20-9-2018	Biological	SMA
**enfortumab vedotin**	Padcev	13-4-2022	Small molecule	SMA
**entrectinib**	Rozlytrek	31-7-2020	Small molecule	CMA
**enzalutamide**	Xtandi	21-6-2013	Small molecule	SMA
**eribulin**	Halaven	17-3-2011	Small molecule	SMA
**fedratinib**	Inrebic	8-2-2021	Biological	SMA
**gemtuzumab ozogamicin**	Mylotarg	19-4-2018	Small molecule	SMA
**gilteritinib**	Xospata	24-10-2019	Small molecule	SMA
**glasdegib**	Daurismo	26-6-2020	Small molecule	SMA
**ibrutinib**	Imbruvica	21-10-2014	Small molecule	SMA
**idecabtagene vicleucel**	Abecma	18-8-2021	CGT	CMA
**idelalisib**	Zydelig	18-9-2014	Biological	SMA
**inotuzumab ozogamicin**	Besponsa	29-6-2017	Biological	SMA
**ipilimumab**	Yervoy	13-7-2011	Biological	SMA
**isatuximab**	Sarclisa	30-5-2020	Small molecule	SMA
**ixazomib**	Ninlaro	21-11-2016	Small molecule	CMA
**larotrectinib**	Vitakvi	19-9-2019	Small molecule	CMA
**lenvatinib**	Lenvima	28-5-2015	Small molecule	SMA
**lisocabtagene maraleucel**	Breyanzi	4-4-2022	CGT	SMA
**loncastuximab tesirine**	Zynlonta	20-12-2022	Small molecule	CMA
**lorlatinib**	Lorviqua	6-5-2019	Small molecule	CMA
**midostaurin**	Rydapt	18-9-2017	Biological	SMA
**mogamulizumab**	Poteligeo	22-11-2018	Biological	SMA
**mosunetuzumab**	Lunsumio	3-6-2022	Biological	CMA
**moxetumomab pasudotox**	Lumoxiti	8-2-2021	Biological	AEC
**necitumumab**	Portrazza	15-2-2016	Small molecule	SMA
**neratinib**	Nerlynx	31-8-2018	Small molecule	SMA
**nintedanib**	Vargatef	21-11-2014	Small molecule	SMA
**niraparib**	Zejula	16-11-2017	Biological	SMA
**nivolumab**	Opdivo	19-6-2015	Biological	SMA
**nivolumab**	Nivolumab BMS	20-7-2015	Biological	SMA

**nivolumab/ relatlimab**	Opdualag	15-9-2022	Biological	SMA
**obinutuzumab**	Gazyvaro	23-7-2014	Small molecule	SMA
**olaparib**	Lynparza	16-12-2014	Biological	SMA
**olaratumab**	Lartruvo	9-11-2016	Small molecule	CMA
**osimertinib**	Tagrisso	2-2-2016	Small molecule	CMA
**padeliporfin**	Tookad	10-11-2017	Small molecule	SMA
**palbociclib**	Ibrance	9-11-2016	Small molecule	SMA
**panobinostat**	Farydak	28-8-2015	Biological	SMA
**pembrolizumab**	Keytruda	17-7-2015	Small molecule	SMA
**pemigatinib**	Pemazyre	26-3-2021	Biological	CMA
**pertuzumab**	Perjeta	4-3-2013	Small molecule	SMA
**pixantrone**	Pixuvri	10-5-2012	Biological	CMA
**polatuzumab vedotin**	Polivy	16-1-2020	Small molecule	CMA
**pomalidomide**	Imnovid	5-8-2013	Small molecule	SMA
**ponatinib**	Iclusig	1-7-2013	Small molecule	SMA
**pralsetinib**	Gavreto	18-11-2021	Biological	CMA
**ramucirumab**	Cyramza	19-12-2014	Small molecule	SMA
**regorafenib**	Stivarga	26-8-2013	Small molecule	SMA
**ribociclib**	Kisqali	22-8-2017	Small molecule	SMA
**ripretinib**	Qinlock	18-11-2021	Small molecule	SMA
**rucaparib**	Rubraca	24-5-2018	Small molecule	CMA
**ruxolitinib**	Jakavi	23-8-2012	Biological	SMA
**sacituzumab govitecan**	Trodelvy	22-11-2021	Small molecule	SMA
**selinexor**	Nexpovio	26-3-2021	Small molecule	CMA
**selpercatinib**	Retsevmo	11-2-2021	Small molecule	CMA
**sonidegib**	Odomzo	14-8-2015	Small molecule	SMA
**sotorasib**	Lumykras	6-1-2022	Small molecule	CMA
**tafasitamab**	Minjuvi	26-8-2021	Biological	CMA
**tagraxofusp**	Elzonris	7-1-2021	Biological	AEC
**talazoparib**	Talzenna	20-6-2019	Small molecule	SMA
**talimogene laherparepvec**	Imlygic	16-12-2015	CGT	SMA
**tebentafusp**	Kimmtrak	1-4-2022	Biological	SMA
**teclistamab**	Tecvayli	23-8-2022	Biological	CMA
**tegafur/gimeracil/ oteracil**	Teysuno	14-3-2011	Small molecule	SMA
**tepotinib**	Tepmetko	16-2-2022	Small molecule	CMA
**tisagenlecleucel**	Kymriah	23-8-2018	CGT	SMA
**tivozanib**	Fotivda	24-8-2017	Small molecule	SMA
**trametinib**	Mekinist	30-6-2014	Small molecule	SMA
**trastuzumab deruxtecan**	Enhertu	18-1-2021	Biological	CMA

**trastuzumab emtansine**	Kadcyla	15-11-2013	Biological	SMA
**trifluridine/ tipiracil**	Lonsurf	25-4-2016	Small molecule	SMA
**tucatinib**	Tukysa	11-2-2021	Small molecule	SMA
**vandetanib**	Caprelsa	17-2-2012	Small molecule	CMA
**vemurafenib**	Zelboraf	17-2-2012	Small molecule	SMA
**venetoclax**	Venclyxto	5-12-2016	Small molecule	CMA
**vismodegib**	Erivedge	12-7-2013	Small molecule	CMA
**zanubrutinib**	Brukinsa	22-11-2021	Small molecule	SMA

[a] Full name: Allogeneic T cells genetically modified with a retroviral vector encoding for a truncated form of the human low affinity nerve growth factor receptor (ΔLNGFR) and the herpes simplex I virus thymidine kinase (HSV-TK Mut2).

Abbreviations: AEC: authorisation under exceptional circumstances, CGT: cell and gene therapy, CMA: conditional marketing authorisation, SMA: standard marketing authorisation
